# St18 specifies globus pallidus projection neuron identity in MGE lineage

**DOI:** 10.1038/s41467-022-35518-5

**Published:** 2022-12-14

**Authors:** Luke F. Nunnelly, Melissa Campbell, Dylan I. Lee, Patrick Dummer, Guoqiang Gu, Vilas Menon, Edmund Au

**Affiliations:** 1 Department of Pathology & Cell Biology, Columbia University Irving Medical Center, New York, NY 10032 USA; 2 Department of Neurology, Columbia University Irving Medical Center, New York, NY 10032 USA; 3 Department of Cell and Developmental Biology, Vanderbilt University School of Medicine, Nashville, TN 37232 USA; 4 Department of Rehabilitation and Regenerative Medicine, Columbia University Irving Medical Center, New York, NY 10032 USA; 5 Columbia Translational Neuroscience Initiative Scholar, Columbia University Irving Medical Center, New York, NY 10032 USA

**Keywords:** Differentiation, Cell fate and cell lineage, Cell migration

## Abstract

The medial ganglionic eminence (MGE) produces both locally-projecting interneurons, which migrate long distances to structures such as the cortex as well as projection neurons that occupy subcortical nuclei. Little is known about what regulates the migratory behavior and axonal projections of these two broad classes of neurons. We find that St18 regulates the migration and morphology of MGE neurons in vitro. Further, genetic loss-of-function of St18 in mice reveals a reduction in projection neurons of the globus pallidus pars externa. St18 functions by influencing cell fate in MGE lineages as we observe a large expansion of nascent cortical interneurons at the expense of putative GPe neurons in St18 null embryos. Downstream of St18, we identified Cbx7, a component of Polycomb repressor complex 1, and find that it is essential for projection neuron-like migration but not morphology. Thus, we identify St18 as a key regulator of projection neuron vs. interneuron identity.

## Abstract (web summary)

The medial ganglionic eminence produces both interneurons and projection neurons, though how this fate choice is made is not well established. Here they show that St18 regulates migration and morphology of MGE neurons, inducing projection neuron fates.

## Introduction

The medial ganglionic eminence (MGE) is a neurogenic progenitor zone that gives rise to both short-range interneurons and long-range projection neurons that distribute broadly throughout the telencephalon^1–3^. Interneuron versus projection neuron is one of the fundamental organizing principles of nervous system architecture, representing local versus point-to-point flows of information, respectively. However, the means by which multipotential neural stem cells in the MGE, and indeed elsewhere in the vertebrate nervous system, delineate interneurons from projection neuron cell fate is poorly understood. While the MGE is best known for generating the majority of the GABAergic interneurons in the cortex, it also produces inhibitory neurons that populate the hippocampus and striatum as well as subcortical nuclei, including the globus pallidus pars externa (GPe) and medial amygdala (MeA)^1–6^. MGE neural progenitors are defined by their expression of the transcription factor Nkx2-1^7–9^. However, as neural progenitors progress through and shortly after they exit the cell cycle, recent single-cell RNA-seq (scRNAseq) studies have found that immature MGE neurons begin to express distinct gene repertoires en route to terminal differentiation^10,11^. A key challenge is to link the expression of these early transcriptional antecedents with mature neuronal identity.

Two of the cardinal features of neuronal specification in the central nervous system are migratory behavior and the type of axon projection. During development, MGE neurons adopt distinct migratory routes. Cortical interneurons, for instance, migrate long distances through a process known as tangential migration in order to reach the neocortex^12^. MGE-derived projection neurons, by contrast, need only migrate short distances from their subpallial site of origin to contribute to subcortical nuclei including the basal ganglia^5^. Once neurons arrive in their appropriate location, interneurons and projection neurons elaborate morphologies appropriate for their function: Interneuron axonal and dendrite arbors project and branch locally, while the axons of projection neurons extend long distances.

The MGE generates subcortical projection neuronal lineages that contribute to the MeA and GPe. While much is known about MGE production of cortical interneurons^12^, the process by which MGE progenitors generate its various projection neuron lineages is still poorly understood. In the GPe, the majority of MGE-derived neurons are called prototypic neurons that express parvalbumin (PV) and primarily project caudally to the subthalamic nucleus (STN). They constitute a key link in the indirect pathway of the basal ganglia^13,14^. In this circuit, the GPe receives inhibitory input from the striatum and projects to the STN where it delivers high frequency, tonic inhibitory tone^15–17^.

A set of factors that could potentially regulate MGE development is the Myelin Transcription Factors (Myt TFs) gene family. They include three paralogs, Myt1 (Nzf2), Myt1L (Myt2 or Nzf1), and St18 (Nzf3 or Myt3) that are highly expressed in the progenitor cells of the neural and neuroendocrine lineages^18–20^. They can either activate or repress transcription depending on cellular context^21^. In humans, Myt TF mutations have been associated with cancer^22^, neuronal dysfunction^23,24^, and neuroendocrine abnormalities^25,26^. In model organisms, Myt1 and Myt1L were reported to regulate endocrine/neuronal differentiation or trans-differentiation by repressing non-neuronal genes^19,27^, while various MyT family members, including St18 regulate pancreatic islet cell function and survival^28–30^. The function of Myt1L and St18 in vivo during neural differentiation, however, remains largely unexplored.

In this work, we show that St18 directs two aspects of projection neuron identity: migratory behavior and axonal morphology using an ES cell-based model of MGE development^31^. Genetic loss of function of *St18* reveals a specific reduction in GPe prototypic neurons, a projection neuron population originating from the MGE. scRNA-seq analysis of the MGE indicated that *St18* regulates progenitor cell specification, which manifests as altered neuronal output. Finally, we identified Cbx7 as a downstream effector of *St18*, which regulates migration, while sparing *St18*-mediated projection neuron-like morphology suggesting that St18-mediated neuronal subtype specification is achieved through different downstream molecular modules independently regulating migration and axonal morphogenesis.

## Results

### *St18* is transiently expressed in medial ganglionic eminence

**Fig. 1:**
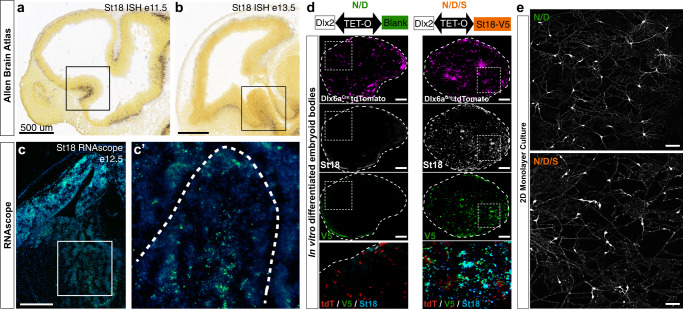
St18 expression in the MGE and St18 GOF in in vitro model of MGE development. **a** E11.5 and **b** E13.5 parasaggital brain in situ for St18 (Allen Brain Atlas). MGE indicated by boxed areas. **c** E12.5 coronal brain RNAscope (green; DAPI blue) for St18, MGE indicated by boxed area, shown in higher power in (**c**’). **d** Schematic of transcriptional GOF in N/D and N/D/S conditions. N/D and N/D/S ES cell lines are differentiated over 12 days into EBs. Sectioned differentiated EBs from N/D and N/D/S shown below schematic with conditional fate-map (Dlx6aCre; Ai9) and immunolabeling for St18 and V5 tag. **e** 2D monolayer cultures from dissociated EBs shown with conditional fate-map (Dlx6aCre; Ai9, white). EBs are gently dissociated and then plated at high density to optimal survival and then cultured for 12 days before imaging. Immunohistochemistry and RNAscope repeated across 3 biological replicates. Scale bars: **a**–**c** 500 microns; **d**, **e** 100 microns.

St18 is a transcription factor transiently expressed in neural progenitors and immature neurons during embryonic development, including expression in the MGE^32^; Allen Institute, Developing Mouse Brain Atlas (Fig. 1a, b). We first confirmed embryonic expression of St18 by RNAscope. At E12.5, we found that St18 transcript is present in the MGE subventricular zone (SVZ) and mantle (Fig. 1a). We next investigated St18 expression by immunohistochemistry. At E13.5, we found that St18 protein is present in the MGE SVZ and mantle and is co-localized with MGE lineage neurons fate-mapped^3^ with a conditional tdTomato reporter (*Nkx2-1*^Cre^; Ai9) (Figure S1b1, b2) but was largely absent in tangentially migrating MGE interneurons (Figure S1b3). As with RNAscope, St18 protein is also present in the marginal zone/superficial cortex (Figure S1a3, b3). To validate our antibody, we tested embryonic brain tissue from *St18* (−/−) (whole animal *St18* null) and MGE-specific conditional knockout mice and compared to WT positive controls (Figure S1c–e). Whole tissue *St18* (−/−) animals were generated by crossing an *St18* conditional allele (cKO) to a germline driver line (*E2a*^Cre^), and MGE-specific cKO animals were generated by crossing the *St18* cKO allele to an MGE-specific driver line (*Nkx2-1*^Cre^). Histology for St18 revealed that St18 signal was absent throughout *St18* (−/−) embryos (Figure S1d, e). These findings are consistent with previous work characterizing the *St18* conditional allele and antibody^29,33^. Taken together, our data is consistent with previously reported in situ hybridization data^32^ Allen Institute, Developing Mouse Brain Atlas, and indicates that the genetic knockout strategy to ablate *St18* works as anticipated.

### St18 alters migration and morphology of MGE neurons

To test the function of St18 in the MGE, we employed an in vitro ES cell differentiation model of MGE development, which we previously developed as a tool to systematically test transcription factors expressed in the MGE^31,34^. Briefly, we start with a parental ES reporter line (Dlx6a-Cre; Ai9^35^) that fate maps subpallial neuronal lineages. The line is sequentially transcriptionally specified by Nkx2-1 and Dlx2. Nkx2-1 imparts MGE identity^7–9^ while Dlx2 further specifies GABAergic neuronal lineages^36–38^. The system makes use of a bidirectional Tet-O cassette that allows us to test candidate transcription factors in gain of function alongside Nkx2-1 and Dlx2 (Fig. 1d). Using this system, we generated two lines: one control line for generic GABAergic MGE lineages using Nkx2-1 and Dlx2, which we named N/D, and a second line with Nkx2-1, Dlx2, and St18 (with a V5 tag), named N/D/S, to test how St18 impacts MGE neuronal output.

To verify St18 induction in the N/D/S line, we tested St18 and V5 expression by immunohistochemistry in N/D and N/D/S lines differentiated into embryoid bodies (EBs) and found co-localized labeling for St18 and V5 in the N/D/S line (Fig. 1d). In contrast, St18 was detected only sparsely with an absence of V5 expression in N/D EBs (Fig. 1d). Further, tdTomato induction was not grossly affected by St18 induction (Fig. 1d). Next, we differentiated N/D and N/D/S EBs and dissociated them into monolayer cultures to examine tdTomato+ neurons. Here, we noticed a dramatic change: tdTomato fate-mapped N/D neurons were multipolar with complex morphologies reminiscent of interneurons, and consistent with our previous studies^31,34^ while tdTomato+ N/D/S neurons appeared to have less complex morphologies and bore longer processes, similar to projection neurons (Fig. 1e).

**Fig. 2:**
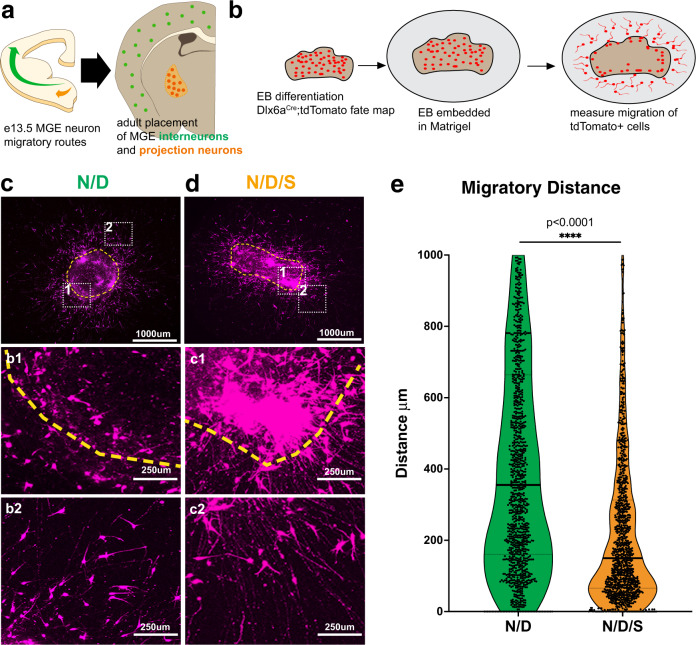
St18 GOF in vitro alters migratory behavior of MGE neurons. **a** Schematic representation (left) of MGE lineage neurons that adopt divergent migratory paths en route to (right) their final position in the adult brain. Cortical interneurons in green, globus pallidus neurons in orange. **b** Experimental design for Matrigel embedding experiments. N/D and N/D/S lines are differentiated into EBs for 12 days after which they are embedded in Matrigel. Fate-mapped neurons (Dlx6a^Cre^; Ai9) are allowed to migrate radially into Matrigel for 4 days before quantification. **c** Representative image of N/D EB with Ai9 tdtTomato in magenta. Yellow dashed line indicates edge of EB. Insets show EB edge (1) and migrating fate-mapped neurons (2). **d** Representative image of N/D/S EB with Ai9 tdtTomato in magenta. Yellow dashed line indicates edge of EB. Subsets show neurons failing to migrate out of EB (1) and elaborated long processes (2). **e** Quantification of neuronal migration in (**b**) and (**c**): Migratory Distance (2-sided Mann–Whitney U Test; *p* < 1e−15). Dataset represents cells assayed from 6 EBs per condition; 1137N/D/S cells and 1156 N/D cells. Source data are provided as a Source data file. Immunohistochemistry repeated across 6 biological replicates.

The MGE generates both short-range interneurons as well as long-range projection neurons^2,3^. Our data indicated that St18 may direct MGE progenitor towards projection neuron identity at the expense of interneuron identity. To test this hypothesis, we more carefully examined N/D/S neurons and N/D neurons by two key features that delineate interneuron and projection neurons: migration and morphology. A defining characteristic of MGE-derived interneurons is their long-distance tangential migration from their birthplace in the ventral telencephalon into the cortex^39^. In contrast, MGE-derived projection neurons migrate shorter distances, where they remain subcortical and populate nuclei in the basal ganglia and amygdala^3–6,40^ (Fig. 2a). Thus, if St18 instructs MGE progenitors to become projection neurons, we would expect that N/D/S neurons will migrate less than N/D neurons. To test this, we embedded N/D and N/D/S EBs in Matrigel and measured the capacity of *Dlx6a*^Cre^; Ai9 fate-mapped neurons to migrate out of the EB (Fig. 2b). Using this assay, we found that N/D neurons robustly migrate out of EBs long distances radially into the Matrigel (Fig. 2c). Conversely, N/D/S neurons migrate shorter distances than N/D controls (Fig. 2d, e), oftentimes failing to leave the EB altogether (Fig. 2d). Further, numerous N/D/S neurons that failed to migrate out bore long processes that emanated from the EB whereas migratory N/D neurons had leading unipolar and branched processes reminiscent of migratory interneurons (Fig. 2c, d, inset 2).

**Fig. 3:**
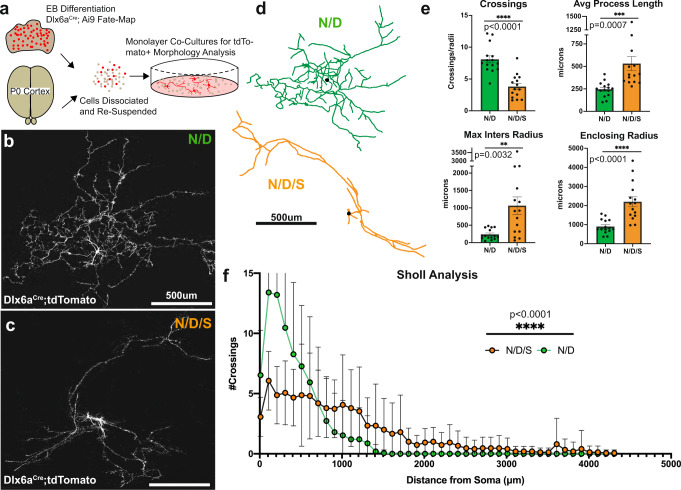
St18 GOF produces projection-like neuronal morphologies. **a** Experimental design of 2D monolayer tracing experiments. N/D and N/D/S lines are differentiated into EBs and dissociated into single-cell suspensions. Concordantly, P0 corticies are dissociated and combined with low-density EB suspensions for plating and whole-cell tracing following fate-mapping (Dlx6a^Cre^; Ai9). **b** Sample N/D neuron plated at low density with fate-mapping (Dlx6a^Cre^; Ai9). **c** Sample N/D/S neuron plated at low density with fate-mapping (Dlx6a^Cre^; Ai9). Morphologcial differences noticeable throughout dataset, see Figure S2. **d** Representative tracings of N/D and N/D/S neurons. **e** Sholl Crossings, Process Length, Maximum Intersection Radius, and Enclosing Radius quantification (Unpaired t-test; Crossings *p* = 0.00000316; Enclosing Radius *p* = 0.0000638). **f** Sholl analysis curves for tracings in (**d**) and (**e**) (two-way ANOVA). Dataset represents 15 reconstructed neurons obtained from 4 separate differentiations per line. Data are presented as mean +/− SEM. Source data are provided as a Source data file.

This prompted us to examine the morphologies of N/D and N/D/S neurons in greater detail. To do so, we dissociated N/D and N/D/S EBs and plated them sparsely onto cortical feeders. This allows for long-term 2D culture of tdTomato+ neurons, which can be analyzed morphologically in isolation (Fig. 3a). We found that N/D/S neurons bear far longer primary processes as well as longer average process length when compared with N/D neurons (Fig. 3b, c, f). Next, we reconstructed N/D and N/D/S neurons and assayed morphological complexity by Sholl analysis and found that N/D/S neurons are significantly less branched with longer, less complex processes compared to N/D neurons (Fig. 3e, f; additional morphological reconstructions in Figure S2). Thus, we find that N/D/S neurons are less migratory than N/D neurons and that N/D/S neurons exhibit a projection-like morphology (less branched with longer processes) that are significantly different from the multipolar, shorter, complex morphologies of N/D neurons. Taken together, St18 regulates neuronal migration and morphology and suggests that it is involved in specifying MGE projection neuron lineages.

### Loss of PV + GPe prototypic projection neurons in *St18* mutants

Given our in vitro findings, we next investigated whether *St18* is necessary for the development of MGE projection neurons in vivo. We first tested if loss of St18 resulted in alterations in MGE proliferation and cell death by analyzing Ki67 and Caspase 3 in *St18* (−/−) embryos, respectively. No significant differences were observed in MGE cells for Ki67 or Caspase 3 in *St18* (−/−) embryos compared to WT controls (Figure S3a-b). We next tested whether *St18* (−/−) embryos exhibited defects in early MGE patterning Nkx2-1+ cells in either the MGE VZ/SVZ or ventrally in the developing basal ganglia (BG)^1^. We found that there is no difference in either the MGE or GP between *St18* (−/−) and WT embryos (Figure S3c). Together, we conclude that *St18* loss of function does not produce changes in proliferation, cell-death, or early MGE patterning. These findings are consistent with our observation that St18 expression is primarily found in SVZ and mantle regions.

**Fig. 4:**
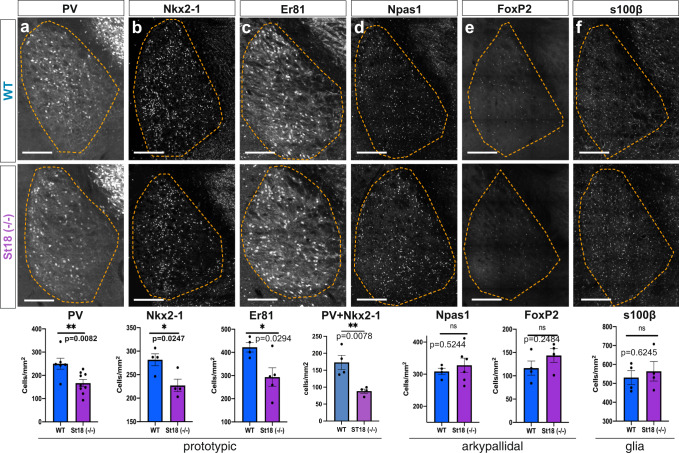
Whole animal St18 KO produces a specific loss of MGE lineage GPe projection neurons. **a** Quantification of PV+ neurons (Unpaired t-test). *N* = 6 WT and 9 *St18* (−/−). **b** Quantification of Nkx2-1+ neurons (Unpaired t-test). *N* = 4 WT and 4 *St18* (−/−). **c** Quantification of Er81+ neurons (Unpaired t-test). *N* = 4 WT and 5 *St18* (−/−). Quantification of Nkx2-1/PV double-positive (Unpaired t-test). *N* = 4WT and 4 St18 (−/−). **d** Quantification of Npas1+ neurons (Unpaired t-test). *N* = 4 WT and 6 *St18* (−/−). **e** Quantification of FoxP2+ neurons (Unpaired t-test). *N* = 4 WT and 4 *St18* (−/−). **f** Quantification of s100b + glial cells (Unpaired t-test). *N* = 4 WT and 4 *St18* (−/−). All data assessed by two-sided Data are presented as mean +/− SEM. Source data are provided as a Source data file. Scale bar represents 500 microns.

The MGE produces GABAergic neurons that populate the cortex, hippocampus, striatum, medial amygdala (MeA), and globus pallidus pars externa (GPe)^3^. We surveyed the MGE neuronal lineage by immunohistochemistry, comparing *St18* (−/−) animals and littermate controls (Figure S4 and Fig. 4). We examined PV+ and SST + neurons in the cortex, hippocampus, and striatum. We also tested the MGE component of the MeA, labeled by SST+ and FoxP2+^41–43^. In the GPe, we counted the number of MGE-derived prototypic neurons, determined by PV, Nkx2-1, and Er81 expression, the number of LGE-derived arkypallidal neurons, determined by Npas1 and FoxP2 expression, and GPe astrocytes, determined by S100b^5,40,44,45^. We found that there was a significant decrease in PV+, Nkx2-1+, and Er81+ prototypic neurons in the GPe in *St18* (−/−) animals compared to controls (Fig. 4). Importantly, we observed no significant difference in any other neural or glial component of the GPe, nor did we observe any significant difference in the other MGE-derived neural lineages throughout the brain (Fig. 4, Figure S4). Indeed, there were no changes in either the somatostatin (SST) or the PV neural populations in the cortex, hippocampus, or striatum following *St18* ablation, nor were there any changes in the SST and FoxP2 MGE-derived neural lineages in the MeA (Figure S4). Thus, we only observe a specific reduction in PV + GPe neurons in *St18* (−/−) animals.

To investigate *St18* genetic ablation with greater resolution, we crossed the *St18* conditional allele to the MGE-specific driver line, *Nkx2-1*^Cre^ and the Ai9 conditional tdTomato reporter. This genetic strategy specifically ablates *St18* in the MGE while simultaneously fate-mapping the MGE neuronal lineage (*St18* cKO). We compared *St18* cKO (St18^fl/fl^; *Nkx2-1*^Cre^; Ai9) to *St18* heterozygote controls (St18^fl/+^; Nkx2-1^Cre^; Ai9). Efficient recombination of the St18 conditional allele in the MGE was confirmed by St18 immunohistochemistry (Figure S1e). We then quantified the number of fate-mapped tdTomato+ neurons throughout the brain to examine MGE lineages in *St18* cKO animals compared with controls. The number of tdTomato+ neurons in *St18* cKO animals was not significantly different in any region other than the GPe (Figure S5), where there was a significant decrease in tdTomato+ neuron density that was not due to a change in total GPe area (Figure S5a). Our analysis of WT vs. cKO cortical interneurons may have been confounded by differences between mutant and control brain size. We therefore measured cortical thickness between the two groups and found no significant differences (Figure S5b). Thus *St18* cKO, consistent with our findings in the *St18* (−/−), exhibits a specific loss of MGE lineage tdTomato+ fate-mapped neurons in the GPe.

**Fig. 5:**
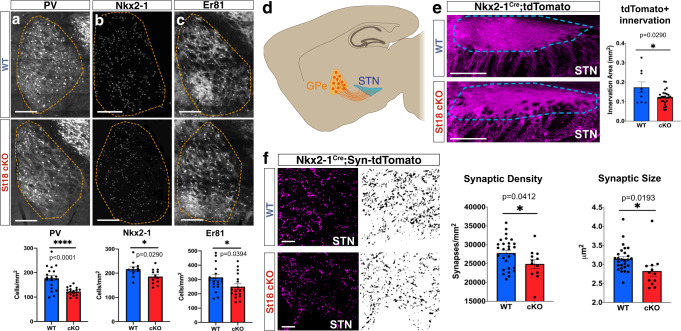
Conditional St18 KO produces a specific loss of MGE lineage GPe projection neurons. **a** Quantification of PV + neurons (Unpaired t-test). *N* = 19 WT and 18 St18 cKO. **b** Quantification of Nkx2-1+ neurons (Unpaired t-test). *N* = 11 WT and 12 *St18* cKO. **c** Quantification of Er81+ neurons (Unpaired t-test). *N* = 20 WT and 18 *St18* cKO. **d** Schematic representation of adult mouse brain in the parasagittal plane showing axonal projections of GPe prototypic neurons (orange) targeting the STN (blue). **e** WT and *St18* cKO STN (outlined in blue) with MGE lineage whole cell labeled fate map (Nkx2-1^Cre^; Ai9). Quantification of axonal innervation area (Unpaired t-test). *N* = 9 WT and 27 *St18* cKO. **f** WT and *St18* cKO STN with (left) MGE lineage synaptophysin labeled fate map (Nkx2-1^Cre^; Ai34). tdTomato, (right) 3D segmented puncta quantified for puncta density and puncta size (Unpaired t-test). *N* = 27 WT and 12 *St18* cKO. Data are presented as mean +/− SEM. Source data are provided as a Source data file. Scale bars: **a**–**c**, **e** 500 microns; **f** 100 microns.

We next repeated our histological analysis of the neural and glial components of the GPe in *St18* cKO. We found that, as in the *St18* (−/−) animal analysis, MGE-derived prototypic neurons, identified by PV, Er81, and Nkx2-1 expression, were reduced while the other neuronal and glial components, determined by Npas1, FoxP2, and S100β, are spared in St18 cKO (Fig. 5a–c; Figure S6). Furthermore, like in *St18* (−/−) animals, *St18* cKO produces no significant change in the MGE-derived neural lineages of the cortex, hippocampus, striatum, and MeA (Figure S7). Taken together, *St18* cKO phenocopies the *St18* (−/−), producing a specific loss of PV + projection neurons in the GPe.

### Disruptions in indirect pathway connections in St18 cKO mice

PV + prototypic neurons in the GPe are an essential component of the basal ganglia indirect pathway. Prototypic neurons of the GPe project caudally to the subthalamic nucleus (STN) (Fig. 5d), which then projects to motor thalamus and the spinal cord to regulate movement^46^. We tested whether loss of PV + GPe neurons in St18 cKO animals resulted in a loss of innervation to the STN. The Ai9 reporter fills axonal processes, allowing us to examine projections from fate-mapped prototypic GPe neurons to their target in the STN. We found a significant decrease in tdTomato+ axons in the STN in St18 cKO animals compared to controls (Fig. 5e). To visualize synaptic innervation directly, we utilized a different reporter line, Ai34, that conditionally expresses synaptophysin-tdTomato (syn-tdT) upon Cre recombination. Syn-tdT puncta were segmented and quantified in the STN, revealing that puncta density and average puncta size were significantly reduced in *St18* cKO (Fig. 5f). As a control, we examined Ai34 puncta density in the dorsal striatum and the GPe, regions that are not primary targets of PV + prototypic neurons in the GPe and found no significant differences (Figure S6d, e). Taken together, *St18* cKO results in reduced innervation of STN due to reduced axonal projections, synaptic density as well as reduced synaptic puncta size, all of which track with a reduction of PV + prototypic neurons present in the GPe.

### Alterations in neuronal output in St18 mutants

Given the highly specific reduction in GPe prototypic projection neurons observed in *St18* mutants, we next examined how cell loss occurs. We found that overall proliferation, cell death, and Nkx2-1+ progenitors were not significantly altered in the mutant MGE (Figure S3). We therefore explored the possibility that *St18* directs the cell fate of GPe prototypic neurons during progenitor cell specification. To do so, we performed single-cell RNA-seq (scRNA-seq) on e14.5 *St18* (−/−) and WT littermate control MGE (*n* = 2 biological replicates per group; median of ~9500 cells passing quality control per biological replicate). We chose to examine *St18* (−/−) instead of *St18* cKO MGE to guard against incomplete recombination with *Nkx2-1*^Cre^, which is unable to recombine in the MGE/LGE sulcus region^47^. We found that St18 transcript was still present in St18 (−/−) samples, although at significantly lower levels in neurons (Figure S10a; *p* = 9.184 × 10^−5^; Wilcoxon rank-sum test). This finding is consistent with previous work describing the *St18* conditional allele in that the transcript is still detectable after recombination, but at lower levels and likely reflects a degree of nonsense-mediated decay^29,33^. Importantly, and consistent with previous work^29^, we found a lack of St18 protein signal in the *St18* (−/−) embryo by immunohistochemistry using a protein that recognizes the N-terminus (Figure S1). Thus, the N-terminal fragment of St18 that is encoded by the recombined St18 transcript is not detectable, likely due to protein instability.

**Fig. 6:**
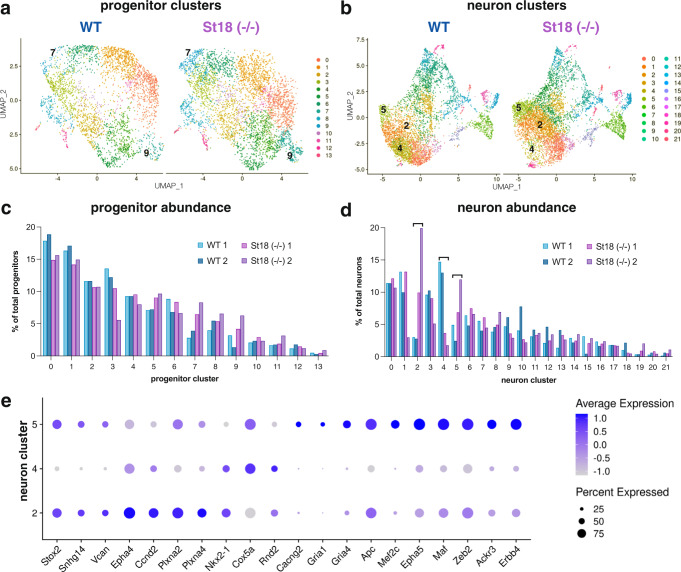
Single-cell RNA-seq reveals that specific neuronal clusters are differentially produced upon St18 genetic ablation. **a** UMAP visualization of 14 progenitor clusters identified in WT (left) and *St18* (−/−) (*St18* (−/−), right) e14.5 MGE. **b** UMAP visualization of 22 neuronal clusters identified in WT (left) and *St18* (−/−) e14.5 MGE. Relative proportions of each **c** progenitor cluster and **d** neuronal clusters, biological replicates plotted separately. WT replicates 1 and 2 in shades of blue, *St18* (−/−) replicates 1 and 2 in shades of purple. **e** Dot plot showing scaled average expression of differentially-expressed genes in neuron clusters 2, 4, and 5. Numbers indicated on UMAP visualizations in (**a**) and (**b**) denote clusters with the most pronounced alterations in proportional representation between WT and *St18* (−/−). scRNA-seq dataset represents 2 biological replicates per genotype, 8143 WT cells and 9922 St18 (−/−) cells that passed quality control total.

Sequencing data were analyzed in a similar manner to a previous study of MGE progenitors and neurons^11^. After filtering cells with fewer than 200 genes detected, we performed a standard clustering analysis using the Seurat package in R^48^. Briefly, we identified high variance genes, followed by principal component analysis (retaining 49 PCs) and Louvain-based community detection clustering. Following subclustering of neurons and progenitors, we identified 14 progenitor cell clusters (3039 total cells in WT, 4018 total cells in *St18* (−/−)) and 22 neuronal clusters (5104 total cells in WT, 5904 total cells in *St18* (−/−)) (Fig. 6a, b; Figure S9). A UMAP visualization of progenitor cell clusters in WT and *St18* (−/−) samples indicate that there are small differences in the relative proportions of clusters between the two groups, with a potential increase in progenitor clusters 7 and 9 in *St18* (−/−) (Fig. 6a, c). However, a similar visualization of neuronal clusters shows more pronounced alterations in neuronal representation; indeed, statistical analysis using a Fisher exact test identified that the proportion of cluster 4 neurons, as a fraction of total neurons, was reduced 4.72-fold (*p* = 3.00 e^−103^), while the proportion of cluster 2 neurons was increased 4.74-fold (*p* = 1.39 e^−99^), and the proportion of cluster 5 neurons was increased 2.35-fold (*p* = 3.12 e^−28^) in *St18* (−/−) versus WT (Fig. 6b, d). Analysis of gene expression in cluster 2 and 5 neurons revealed enrichment for cortical interneuron-related genes, for example: *Maf* (*c-Maf*)^48–51^, *Zeb2* (*Zfhx1b*, *Sip1*)^49,52^, *EphA4*^53^, *Ackr3* (*CxcR7*)^54,55^, and ErbB4^56^ over WT cluster 4 neurons (Figure S6e). St18 is not expressed at high levels in neuron clusters 2, 4, or 5 (Figure S10c). Thus, loss of *St18* likely affects progenitor cell production of neurons, resulting in a shift towards interneuron output (clusters 2 and 5) and away from cluster 4, which are likely to be nascent GPe prototypic neurons.

### Cbx7 regulates projection neuron migration downstream of St18

To determine how St18 regulates projection neuron identity in MGE progenitors, we examined differential gene expression between WT and *St18* (−/−) MGE in vivo and then cross-referenced our findings with bulk RNA sequencing (RNA-seq) in N/D and N/D/S neurons in vitro. We found that Cbx7 was one of the most differentially expressed genes in N/D/S line vs. N/D; it was strongly upregulated in the N/D/S line (log_2_FC = 2.09, ~4.25-fold) (Figure S12a).

Cbx7 is a component of the Polycomb repressor complex 1 (PRC1), which broadly enacts transcriptional repression^57^. Cbx7, via its association with the PRC1, is involved in both the early embryonic development of neural tissue^58^ as well as in post-mitotic differentiation of maturing neurons^59^, making it an attractive candidate to investigate as a potential downstream effector. We found Cbx7 in our scRNA-seq analysis, but it was detected at overall low levels. We therefore examined our scRNA-seq dataset for the expression of Cbx7 targets, which had previously been identified by ChIP-seq^60^. We found that the large majority of targets had elevated levels of expression in St18(−/−) vs. WT neurons (Figure S11a). Since we found elevated Cbx7 levels in N/D/S neurons, this is consistent with the notion that, in the absence of St18, Cbx7 targets are de-repressed compared to WT. We also compared Cbx7 targets expression levels for neuron clusters 2, 4, and 5, reasoning that if St18 distinguishes neuron cluster 4 from clusters 2 and 5, Cbx7 targets should correspondingly be expressed at higher levels for 2 and 5 compared to 4. Indeed, this was what we observed for many Cbx7 targets (Supplemental Figure 11b). Taken together, our in vitro and in vivo expression analysis indicated that Cbx7 was a suitable candidate for functional analysis.

**Fig. 7:**
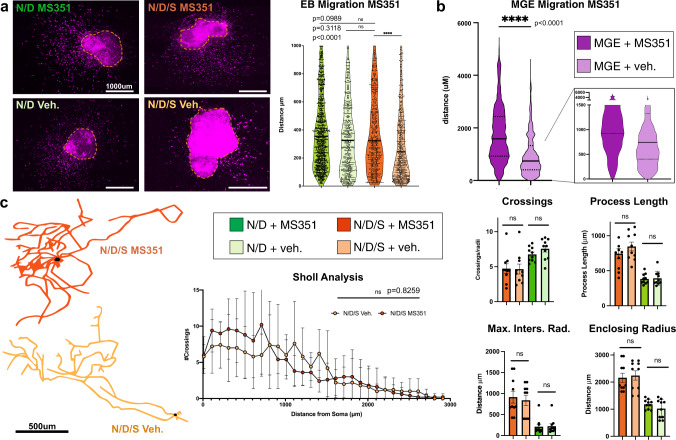
St18 regulates projection neuron migration but not morphology via upregulation of Cbx7. **a** Sample images of Matrigel embedded N/D/S EBs treated either with MS351 or Veh. Ai9 tdtTomato in magenta. Quantification to the right of the images (Mann–Whitney U Test; N/D/S MS351 vs. vehicle *p* = 0.0000146). Dataset represents 3 EBs per condition; 531 N/D/S MS351 cells and 614 N/D/S Veh. cells and 1301 N/D MS351 cells and 567 N/D Veh. cells. **b** MGE explants from Nkx2-1^Cre^; Ai9 e12.5 embryos treated with MS351 or Veh. Quantification of tdTomato+ cell migration from edge of explant. Inset on right emphasizes migration <1500 microns. Dataset represents 3 MGE per condition; ~20,000 cells quantified per group (K-S test; *p* < 1e−15). **c** Sample neuronal reconstructions in N/D/S MS351 and N/D/S Veh. conditions. Sholl analysis (two-way ANOVA) and Sholl statistics quantification to the right of the neuronal reconstructions (Unpaired t-test). *N* = 10 N/D/S MS351 and 10 N/D/S Veh. and 10 N/D MS351 and 10 N/D Veh. Data are presented as mean +/− SEM. Source data are provided as a Source data file.

We utilized a pharmacological inhibitor of Cbx7-mediated repression, MS351^61^ to test if it affected the migratory and morphological phenotypes of N/D/S neurons in vitro. To test the efficacy of MS351, we assayed P16^INK4a^ transcript levels by qPCR^62^ in N/D/S neurons since P16^INK4a^ transcription is negatively regulated by Cbx7-mediated PRC1 activity^63^. We found that N/D/S cells treated with MS351 had higher levels of P16^INK4a^ compared to N/D/S cells treated only with vehicle (ΔΔCt = −3.62; ~150-fold increase MS351-treated vs. vehicle). We found that N/D neurons migrate significantly further than N/D/S in our in vitro migration assay (Fig. 2). However, following application of MS351, N/D/S neurons now migrate significantly further than vehicle-treated N/D/S neurons (Fig. 7a). In fact, MS351-treated N/D/S neurons migrate as far as N/D neurons treated with MS351. Notably, MS351-treated N/D cell migration is not significantly different from vehicle-treated N/D neurons (Fig. 7a). This result suggests that N/D/S neurons treated with MS351 lose projection neuron-like migratory behavior and instead, migrate in an interneuron-like manner. We next tested whether this held true with bona fide MGE neurons (Fig. 7b). When MGE is dissected and cultured in Matrigel, MGE neurons (a mixture of interneurons and projection neurons) migrate out of the MGE explant, a configuration that parallels our experiments with ES-derived EBs in Matrigel. We measured the migratory distance from the edge of the explant of Nkx2-1^Cre^; Ai9 fate-mapped MGE neurons cultured with MS351 or vehicle control. We found that MS351-treated neurons migrated significantly longer distances than vehicle and that this effect was especially pronounced in tdT+ MGE neurons that migrate short distances (Fig. 7b). Possibly, these neurons correspond to MGE projection neurons that possess an intrinsically diminished capacity to migrate compared to interneurons—thus, potentially mirroring our findings with N/D/S and N/D neurons (Fig. 7a). We next tested the effect of MS351 on neuronal morphology. Here, we found that MS351 had no effect on N/D/S or N/D morphologies as assessed by Sholl statistics and Sholl analysis (Fig. 7c, Figure S12b, c). Thus, Cbx7 regulates St18-mediated migration, while sparing St18-mediated projection neuron morphology altogether.

## Discussion

Many neural progenitors throughout the brain generate both interneurons and projection neurons, but how this crucial fate choice is determined is very poorly understood. In this work, we demonstrate that the transcription factor St18 directs MGE progenitors to adopt projection neuron identity at the expense of interneuron production. Using an in vitro assay, we find that St18 governs the migratory and morphological characteristics of ES-derived MGE neurons towards projection neuron identity, overriding the default interneuron fate of N/D cells. In whole animal null and conditional loss-of-function *St18* mutants, we observe a highly specific loss of prototypic GPe projection neurons, which is accompanied by a reduction in target innervation of the STN. By single-cell RNA-seq analysis of *St18* (−/−) and wild-type MGE, we find that while MGE progenitors are largely unaffected, neuronal output is altered in *St18* mutants; specifically, we observe an expansion of nascent interneurons and a decrease in putative GPe prototypic neurons. Gene expression analysis identified Cbx7 as a downstream effector of St18. Through pharmacological inhibition, we show that Cbx7 governs St18-mediated cell migration, while St18-mediated projection neuron morphology is unaffected. We therefore describe a transcriptional determinant in vertebrate brain that directs neural progenitors towards projection neuron over interneuron fate. Downstream of St18, Cbx7 specifically regulates migratory aspects of the projection neuron phenotype, demonstrating that different attributes of MGE projection neuron identity are potentially parsed into distinct transcriptional programs.

Previous studies have identified genes responsible for various characteristics of MGE lineage neurons. These include specification of MGE ventricular progenitors by sustained expression of *Nkx2-1*^64,65^, the regulation of PV cortical interneuron migration by Sp8*/*Sp9^66^, the differentiation and maturation of SST cortical interneurons by *Satb1*^67^, and the allocation of MGE lineage striatal neurons via chemoattractive and chemorepulsive cues in response to Nrg1/ErbB4 and EphB/ephrinB signaling, respectively^68^. However, to our knowledge, St18 is the first transcription factor identified in the vertebrate brain that directs progenitor cells to delineate between projection neuron over interneuron identity.

St18 (also known as Myt3 and NZF3) is a member of the Myt family of transcription factors, characterized by a conserved zinc finger DNA binding motif^32,69^. Other Myt transcription factor superfamily members include Myt1, which promotes neurogenesis via inhibition of Notch signaling^19^, and Myt1L, one of the ‘BAM’ factors required to produce induced neurons^70^ that drives neuronal fate by suppressing non-neuronal differentiation^27^. Further, Myt1 mutant mice exhibit defects in vagal nerve projections^20^. St18 has also been implicated in neuronal differentiation, acting in conjunction with Neurog1^71^, and is thought to act broadly through gene repression due to its high-affinity DNA binding domain. St18 also has a documented role in cancer, acting as a tumor suppressor^22^—hence, its name: suppressor of tumorogenicity 18—or as an oncogene^72^ depending on tissue type. St18 also mediates apoptosis in pancreatic beta cells^28^ and cell migration in islet cells through integrin signaling^73^. The latter is consistent with our finding that St18 regulates migration of MGE neurons in vitro.

Migratory patterns of MGE-lineage neurons are divergent. Cortical interneurons migrate long distances tangentially and then switch to radial migration in order to arealize throughout the cortex. Subpallial MGE neurons, on the other hand, migrate comparatively short distances to occupy the striatum, globus pallidus, and amygdala. While there is much known about factors that govern interneuron tangential migration into the cortex^74^, relatively little is known about short distance migration in subpallial MGE neurons. In fact, many of the known factors for MGE interneuron migration are guidance cue-related^74,75^: Robo/Slit^76^, Eph/ephrin^53,77^, semaphorin^64,78^, Cxcr/Cxcl^54,55^, RGMa-neogenin^79^, and Netrin^80^. Here, we find that St18 induction robustly curtails cell migration in N/D/S neurons versus N/D neurons. Given the paucity of guidance cues present in our in vitro migration assay, we surmise that St18 restricts the cell intrinsic capacity for MGE neurons to migrate long distances, which is a mechanism that is distinct from expression of guidance cues. During development, this restricted migratory capacity likely acts in concert with guidance cues in the orchestrated assembly of the GPe^5^ to ensure the appropriate placement of GPe prototypic neurons during development.

The GPe is comprised of two principle neuronal populations. One population are the arkypallidal cells that are primarily generated in the lateral ganglionic eminence and project back to the striatum. The second population are prototypic cells that are primarily MGE-derived and project mainly to the STN^2,5,40,44^. Recent work describes seven distinct classes of GPe neurons^81^. Among these classes, four are PV + prototypic cohorts, each with different, non-overlapping molecular marker expression and electrophysiological characteristics. Our analysis of St18 (−/−) and *St18* cKO reveal a specific and significant loss of PV + prototypic neurons accompanied by loss of Nkx2-1, Er81, and tdTomato+ cells. We also observe a proportional reduction in fate-mapped synaptic innervation of the STN. Notably, we do not observe a complete loss of GPe prototypic neurons in *St18* mutants, leading us to hypothesize that *St18* is required for the specification of a subset of prototypic neurons and indicates that further work is required to parse the developmental origins of GPe cell types.

Two recent studies examined the dynamic process of MGE neurogenesis by scRNA-seq^10,11^. They find that MGE progenitors share similar gene expression patterns and then rapidly diversify as they transition out of the cell cycle. Indeed, *St18* was identified as a marker gene for an MGE progenitor pool during this process of diversification^11^. However, shortly after MGE neurons are specified, they migrate extensively throughout the brain, making it a difficult task to link developmental gene expression with mature neuronal identity. In our scRNAseq analysis, we find a number of St18-expressing progenitor populations (progenitor clusters 0, 4, 7, 11, and 12; Figure S10b). However, we find that loss of *St18* does not largely affect the relative proportion of MGE progenitors. Instead, we observe sizable alterations in neuronal output, namely the loss of neuron cluster 4 and an expansion of neuron clusters 2 and 5. It is notable, however, that none of the affected neuronal clusters express St18 at high levels. This leads us to hypothesize that *St18* likely exerts its effect on cell identity in progenitors cells as they exit the cell cycle and begin to adopt neuronal identity. This has parallels with the transcription factor that defines MGE identity, Nkx2-1, which continues to influence cell fate up until cell cycle exit in a similar manner^7^.

We note that the specificity of the scRNA-seq phenotype mirrors the specific loss of GPe prototypic neurons in the *St18* mutant. Along this line, it is appealing to speculate that the identity of neuron cluster 4, which is strongly diminished in *St18* (−/−), are nascent GPe prototypic neurons. In support of this, we find that cluster 4 neurons are enriched in expression for *Nkx2-1*, whose sustained expression is found in subcortical MGE populations including GPe prototypic neurons^1,5^. We also detect higher levels of *Rnd2* in cluster 4, which is associated with radial migration^82^. Given the close proximity of the MGE progenitor domain to the final location of the GPe, it is likely that immature GPe neurons migrate via radial versus tangential migration. In contrast, neuron clusters 2 and 5, which are expanded in *St18* (−/−), exhibit higher levels of *APC*, which is associated with tangential migration in cortical interneurons^83^. Indeed, we find numerous genes in clusters 2 and 5 that are reminiscent of cortical interneurons and express markers that indicate that they are nascent PV + cortical interneurons: *Mef2C*^50^ and *Ccdn2*^84^. Thus, in the *St18* (−/−) MGE, we find selective alterations in three PV + neuronal populations; given the reduction of cluster 4, accompanied by the expansions of cluster 2 and 5, we hypothesize that the 3 PV + populations share a common lineal origin that is demarcated by the presence or absence of St18 expression at the progenitor cell stage.

While in *St18* (−/−) we observe robust expansions of neuron clusters 2 and 5, and similarly sharp reduction in neuron cluster 4 in our scRNA-seq analysis, it stands in contrast to the relatively moderate (~35%) reduction in GPe prototypic neurons in *St18* mutants and the lack of an expanded cortical interneuron population. We hypothesize that selective survival and cell death helps normalize cell numbers postnatally, and accounts for the blunted impact observed in adult mutant animals. Certainly, with cortical interneurons, a number of studies demonstrate that their cell numbers are under exquisite regulation^85–88^. It is conceivable that GPe neuronal numbers are similarly self-correcting, but since too few GPe prototypic neurons are produced in the St18 (−/−), selective survival alone is insufficient to bring cell numbers up to appropriate levels.

Very little is known about the molecular regulation of migration and axonal morphogenesis in neurons. To identify factors downstream of *St18* that regulate these two properties of projection neuron identity, we examined gene expression in *St18* (−/−) and wild-type MGE in vivo and cross-referenced with N/D versus N/D/S neuron gene expression in vitro. Through this process, we identified Cbx7, which is strongly enriched in N/D/S neurons and whose gene targets are expressed at aberrant levels in St18(−/−) MGE neurons. Cbx7 (chromobox 7) is a component of the Polycomb repressor complex 1 (PRC1). In this complex, it is involved both in early neural differentiation and post-mitotic neurite extension^59^. Cbx7 is also well-characterized in cancer. Mutations in *Cbx7* are correlated with increased glioblastoma multiforme tumor invasiveness^89,90^. Restriction of migration is thought to be mediated by Cbx7 interaction with Wnt/b-catenin^91^ and the YAP-TAZ pathway^92^. Thus, the elevated levels of Cbx7 observed in decreased cancer metastasis are consistent with our findings that N/D/S neurons express higher levels of Cbx7 and migrate shorter distances.

We made use of a pharmacological inhibitor of Cbx7, MS351, that blocks Cbx7/PRC1-mediated H3K27Me3 transcriptional repression^61^ and found that it resulted in conversion of N/D/S such that they migrate like N/D neurons, while leaving St18-mediated morphology intact. Indeed, we also observed that MS351 administration exerted the same effect on the migratory properties of bona fide MGE neurons in an explant migratory assay. These results raise the intriguing possibility that projection neuron cell identity is adopted in a piece-wise fashion, with migration and morphology being managed by separate transcriptional cascades. If so, this has parallels to what has been termed terminal selector genes in *C. elegans* and *Drosphila*. Terminal selectors are generally post-mitotic transcription factors responsible for directing or maintaining lineage characteristics^93^. For instance, mec-3 directs touch receptor identity in *C. elegans*^94^. In *Drosophila*, Apterous and Islet regulate morphological and physiological features^95,96^. Indeed, some MGE genes such as *Tsc1* that regulates cortical interneuron intrinsic physiology^97^ also regulates specific aspects of interneuron identity. Our results suggest that *St18* and *Cbx7* may function in a similar arrangement, where the former sits atop a genetic hierarchy to direct cell fate while the latter governs gene repression that regulates the migratory portion of projection neuron identity. If this proves to be true, there may also be additional selector-like factors that govern projection neuron morphology and other aspects of GPe prototypic identity. Further work will be required to elucidate whether such an arrangement exists and whether this is a general organizing principle that regulates the cellular properties of other neuronal lineages arising from the MGE and perhaps throughout the brain.

## Methods

### Derivation of N/D and N/D/S lines

We derived a mouse parental ES cell line from a Dlx6a^Cre^ (Jackson Laboratory Stock No. 008199); Ai9 (Jackson Laboratory Stock No. 007909) e3.5 pre-implantation male embryo using previously described methods^98^. To this parental line, we stably transfected 2 transgenes, Nestin-Nkx2-1-IRES-tTA^2^S and either TRE-Dlx2 (N/D) or TRE-Dlx2-St18 (N/D/S), as previously described^31^. Briefly, constructs were linearized and introduced into low-passage parental ES cells on a Nucleofector (Lonza) using the mouse ES cell nucleofection kit (Lonza) using protocol “A24”. Cells were plated at 1:20 density on gelatinized 10 cm plates and grown in ES cell media for 48 h. After this time, hygromycin was added to the media for clone selection. Following 1 week of drug selection, ES cell colonies were clonally selected and expanded on gelatinized 96-well plates and expanded to two plates. One plate was cryopreserved at −80 °C while the other was used for genotyping. Clones were screened by PCR for presence of transgenes. Clones were then expanded and differentiated to screen transgene function before use in experiments. ES cell cultures were maintained and screened using standard practices and protocols^99^ and routinely tested for mycoplasma.

### Specification of ESCs into MGE progenitors

Differentiation of ES cells to become MGE lineage neurons was adapted from protocols previously described^31,34^. Briefly, ES cell cultures were maintained for two passages on 0.1% gelatin (Millipore) using modified Dulbecco’s Modified Eagle Medium (Gibco), 15% heat-inactivated FBS (HyClone), 1x Modified Eagle Medium Non-Essential Amino Acids (Gibco), 1x Sodium Pyruvate (Gibco), and 1x Glutamax (Gibco), 10 µM beta-mercaptoethanol (Fisher Scientific), and 10^4^ U/mL ESGRO LIF (Sigma Millipore). N/D and N/D/S line ESCs were dislodged with 0.025% trypsin/1% EDTA (Gibco), dissociated, and suspended at a density of 50,000 cells/well in a non-TC-treated 24-well plate in 800 µL of pre-mixed differentiation media (Glasgow’s Modified Eagle Medium [Gibco], 1x PenStrep [Gibco], 1x Modified Eagle Medium Non-Essential Amino Acids [Gibco], 1x Sodium Pyruvate [Gibco], and 1x Glutamax [Gibco], 10 µM beta-mercaptoethanol [Gibco], and 4% Knockout Serum Replacement [Gibco]) as described previously^31^. Briefly, cells were neuralized and directed toward telencephalic identity with Wnt-inhibitor XAV-939 (Tocris, 0.1 µM) from days 0–4. Sonic hedgehog agonist (SAG [Tocris], 1 µM) is added to ventralize telencephalic EBs from days 4–6. From day 6 onwards, on SAG is present in the medium until EB differentiation is complete on day 12. Media changes occurred on days 4, 6, and 9 during the protocol, and free-floating embryoid bodies (EBs) containing differentiating neurons form spontaneously during the course of the protocol.

### Embryoid body fixation and histology

Embryoid bodies (EBs) from two rows of a 24-well plate were gathered in a 15 mL conical tube and spun down at 200 × *g* for 2 min, rinsed with PBS and re-spun. EBs were then re-suspended in 1 mL of 4% PFA and incubated for 30 min at room temperature. Post-fixation, EBs were rinsed in PBS and re-suspend in 10% sucrose solution in PBS and incubated for 30 min, then re-suspended in 30% sucrose solution in PBS and incubated for an additional 30 min. Using a P200 wide bore pipette tip, EBs were removed from the tube and placed in the base of a plastic cryomold (Fisher Scientific). Sucrose solution is removed carefully with both a pipette and the folded edge of a Kimwipe until EBs were as dry as possible at the base of the mold. Tissue-Tek OCT (Sakura) is added to the mold and placed on dry ice for flash freezing and then stored at −30 °C. Cryosectioning was performed on a Leica cryostat at a thickness of 15 µm. Sections were permeabilized using a 0.01% Triton X-100 in PBS and blocked overnight with a 5% normal donkey serum (NDS)/PBS solution. Primary antibodies for V5 (Invitrogen Cat. R960-25, 1:200) and St18 (1:1000) were incubated in 5% NDS/PBS overnight at 4 °C and secondary antibodies (Jackson Immunoresearch, 1:1000) diluted in 0.01% Triton X-100/PBS for 2 h at room temperature. Sections were cover slipped with Fluoromount-G (Southern Biotech) and stored at 4 °C prior to imaging with Zeiss epifluourescent microscope at ×200.

### Animals

The conditional *St18* allele was generated by introducing flanking LoxP sites to exons 9 and 10 of *St18*^33^. Cre-mediated recombination causes a frame shift in the coding region and a premature stop codon, resulting in the production of a truncated N-terminal fragment. Using an antibody that recognizes the N-terminus of St18, no signal is detected, likely due to a combination of nonsense-mediated mRNA decay and peptide instability^29^. The *St18*^fl/fl^ mouse is on a mixed C57Bl/6-ICR background. To generate the *St18* null, we crossed *St18*^fl/fl^ with a germline Cre driver (E2a-Cre Jackson Laboratory Stock No. 003314) Experimental null animals were generated from *St18* heterozygote breeders, and wild-type littermates were used as controls. For *St18* conditional knockout (cKO) experiments, the *St18*^fl/fl^ was crossed to an MGE-specific Cre driver (*Nkx2-1*^Cre^ Jackson Laboratory Stock No. 008661) along with the conditional tdTomato reporter (Ai9 Jackson Laboratory Stock No. 007909) or a synaptophysin tagged fate-mapping allele (Ai34 Jackson Laboratory Stock No. 012570) for forward fate-mapping of the *Nkx2-1* lineage. *St18*^fl/+^ breeders were used for *St18* cKO experiments to generate *St18* cKO and heterozygote littermate controls. Using this strategy, we generated St18 cKO mice, St18 WT littermates, and St18 het littermates. A posthoc ANOVA analysis revealed that St18 het cKO animals were statistically the same as WT cKO. We therefore grouped the 2 together as controls for conditional IHC experiments. All animal experiments were conducted in compliance with and pre-approval from Columbia University’s Institutional Animal Care and Use Committee.

### 2D culture and morphometric analysis of ESC derived differentiated neurons

Mouse cortical feeders were prepared from P0-P4 wild-type Swiss-Webster mice. Briefly, cortices were micro-dissected into chilled Hibernate A media (Hibernate A[Gibco], 1% Pen/Strep [Gibco]). 1 mL of a Papain solution of approximately 20 Units/mL was prepared with Hibernate A dissection media and ~1000 units of DNAseI and added to dissected tissue. Cortical tissue is agitated at 37 °C for 15 min. Papain is quenched with 3 mL Hibernate A, after which cortical tissue is triturated with a P1000 pipette tip until fully dissociated (8–10 times). Cells were counted, spun at 200 × *g* for 5 min, and re-suspended in modified Neurobasal A to a concentration of 6 × 10^7^ viable cells per mL, and 50 µL cell suspensions were plated per well. In parallel, EBs were dissociated in 1 mL Papain solution for 15 min, 37 °C with agitation. Cells were then diluted approximately 1/1000 in modified Neurobasal A media in order for labeled cells to be plated at low density for single-cell morphological analysis. The EB/cortical feeder mix is plated onto m 24-well optical plastic tissue culture plates (Ibidi) pretreated with 20 µg/mL poly-D lysine and rinsed thoroughly with sterile water. Neuronal cultures were maintained in a modified Neurobasal A media (500 mL Neurobasal A [Gibco], 0.01 M HEPES [Gibco], 1% PenStrep [Gibco], 1X B27 supplement [Gibco] is added on the day of use). Media is changed on the first day following plating, and every other day subsequent to that before cultures were fixed with 4% PFA in PBS after 12 days of culture. Whole wells were imaged at ×100 magnification using a Zeiss Apotome tiling microscope. Whole neuronal morphologies were traced using the Neuro-Anatomy Simple Neurite Tracing plugin in ImageJ^100^. Morphometric and Sholl analysis were performed on the traces using Neuro-Anatomy plugin (ImageJ). Sholl analysis is performed at a standard radius of 10 mm^101^.

### In vitro migration assay

Individual differentiation day 12 N/D and N/D/S EBs were placed into the center of a 24-well optical plastic tissue culture plates (Ibidi), using a wide-bore P200 pipette tip. Media was gently removed before EBs were suspended in Matrigel (Corning) droplets (50–100 µL) using pre-chilled P1000 pipette tips. EB/droplets were incubated for 30 min at 37 °C. Following incubation, solidified Matrigel droplets containing EBs were suspended in Neurobasal media and cultured for four days until fixation using 4% PFA in PBS. Whole EBs were imaged at ×200 using a Zeiss confocal microscope. Migration distance is analyzed using straight line tool in ImageJ, measuring the distance a cell traveled perpendicular to the edge of the EB.

### RNA preparation and sequencing

Day 12 N/D and N/D/S EBs were collected into a 15 mL conical tube and spun down at 200 × *g* for 5 min. EBs were rinsed with PBS and then suspended in 500 µL 1X AccuMax (Gibco) enzyme solution with 10 Units DNAaseI (Thermo) and incubated for 15 min at 37 °C. The enzyme was quenched with 5 mL HEPES buffered saline and triturated to a single-cell suspension using a P1000 pipette tip. Cells were spun at 300 × *g* for 3 min and re-suspended the pellet in 500 µL HEPES buffered saline. The suspension was filtered with a 70 µm cell strainer for flow cytometry. tdTomato+ cells were sorted (BD Influx System) directly into Trizol. RNA was purified using an RNA miniprep kit (Zymo) and measured with a Bioanalyzer (Agilent) to ensure an RIN score of 7 or greater. mRNA was enriched using a standard poly-A pull-down prior to library construction with Illumina TruSeq chemistry. The library is sequenced using Illumina NovaSeq 6000 at the Columbia Genome Center. Samples were multiplexed in each lane with yields of paired-end 100 bp reads per sample. RTA (Illumina) was used for base calling and bcl2fastq2 (version 2.20) for fastq format conversion. A pseudoalignment was performed to a kallisto index created from the mouse transcriptome using kallisto (0.44.0). We tested for differentially expressed genes using Sleuth, an R package designed for differential gene analysis from kallisto abundance files.

### Single-cell RNA sequencing sample preparation

e14.5 embryos generated from a cross of *St18*^+/−^ x *St18*^+/−^ parents. Embryos were collected and MGE was micro-dissected into chilled Hibernate A solution. During the dissection, embryo DNA was obtained using the HOTSHOT protocol^102^ to allow for rapid PCR genotyping. *St18* null and WT MGE were pooled and dissociated separately. Samples were submitted to the Sulzberger Genome Center Single Cell Core at the Columbia University Medical Center for library construction using 10X Genomics chemistry. Sequencing is run on a 10X Genomics platform, and analysis was performed on 10X Genomics’ Cellranger software version 5.0.1.

### Single-cell RNA sequencing data analysis

After alignment and quantification with Cellranger, cells were filtered based on the following criteria: cells with fewer than 200 unique genes detected, or cells with more than 10% of reads mapping to the mitochondrial genome were excluded from further analysis. After QC, all cells from all experiments were aggregated and clustered using the Seurat package in R, with default parameters, except the following: cell cycle differences among dividing cells were regressed and removed^11^, and clustering was performed on the 49 principal components with a *p*-value less than 0.01, as determined by jackstraw resampling, with a resolution of 1.4, based on bootstrapped iterative clustering^103^. After clustering, progenitor and neuronal clusters were identified by plotting the expression of marker genes from^104^. Subsequently, progenitors and neurons were re-clustered separately. Each cluster was then assessed for statistically significant enrichment or depletion in the WT versus *St18* (−/−) groups, using a Fisher’s exact test with Bonferroni correction for multiple comparisons. This identified three neuronal clusters with statistically significant differences between the groups in terms of proportions, with the additional constraints of comprising >5% of the total neuronal population and at least two-fold change in proportions between the groups. Differential gene expression among the clusters was performed using a Wilcoxon rank-sum test in the Seurat package.

### In vivo tissue preparation and histology

For adult histology, P30-P40 animals older were anesthetized using a cocktail of Ketamine and Xylazine and were sacrificed using a trans-cardiac perfusion of PBS followed by 4% PFA/PBS. Following dissection, brains were post fixed for 24 h in 4% PFA/PBS at 4 °C and then mounted in 4% low melt agarose to prep for vibratome sectioning. Brains were sectioned in the coronal plane at a thickness of 50 µm. Sections were collected in series in 96-well U-bottom plates in an anti-freeze solution (1:1:1 PBS, poly-ethylene glycol, and glycerol) for long-term storage at −30 °C. For immunohistochemistry, free-floating sections containing the areas analyzed (globus pallidus pars externa, medial amygdala, hippocampus, and primary auditory and somatosensory cortices) were permeabilized with 0.01%Triton X-100/PBS. Primary antibodies were incubated for three days at 4 °C in 5% NDS/PBS (PV [ImmunoStar Cat. 24428] 1:1000, Npas1 [Gift from the Chan Lab, Northwestern University] 1:1000, FoxP2 [Gift from the Jessel Lab, Columbia University] 1:1000, Er81 [Gift from the Jessel Lab, Columbia University] 1:32,000, SST [Millipore Sigma Cat. MAB354] 1:250, s100β [Millipore Sigma Cat. SAB5600115] 1:1000, Nkx2-1 [Abcam Cat. Ab227652] 1:500), and secondary antibodies (Jackson Immunoresearch). Rat-anti-St18 antibody was produced in the as described previous^33^. Briefly, the N-terminus of St18 (aa 60–298) was fused to a maltose-binding protein to generate a purified immunogen. Strategic Bio-Solutions (Newark, DE) used the immunogen to generate rat polyclonal anti-St18 antibody.

Sections were mounted by paint brush onto SuperFrost Plus slides in Fluoromount G solution for long-term storage. For embryonic histology experiments, e13.5 timed plugs were prepared using *St18* null heterozygous breeders, and pregnant dams were sacrificed using carbon dioxide euthanasia and cervical dislocation prior to embryo dissection. Embryos were immersion fixed in 4% PFA/PBS for 15 min, rinsed with PBS, before overnight cryoprotection in 30% sucrose/PBS solution. Following cryoprotection, embryos were mounted in OCT compound and flash frozen on dry ice and stored at −30 °C prior to sectioning. Cryosectioning was performed on a Leica cryostat at 15 µm thickness, and sections were dried at room temperature for 1 h before freezing at −30 °C. Immunolabeling was performed following a 20 min wash in PBS to remove excess OCT. Primary antibodies were incubated overnight at 4 °C in 5% NDS/PBS (Nkx2-1 [abcam Cat. Ab227652] 1:500, St18, 1:1000, Ki67 [Invitrogen Cat. 14-5698-82] 1:200, Caspase 3 [Novus Cat. 31A1067] 1:500), and secondary antibodies (Jackson Immunoresearch). Sections were cover slipped in Fluoromount G solution for long-term storage.

### Imaging and image analysis

All imaging was performed at ×200 using either a Zeiss scanning confocal microscope or a Zeiss epifluourescent tiling microscope at specific ROIs based on the Allen Brain Institute Reference Atlas. All adult histology (PV, Npas1, Nkx2-1, Er81, FoxP2, SST, and s100β) and tdTomato analysis was performed following imaging with the Zeiss epifluourescent scope at ×200, and representative images for figures were taken with the Zeiss confocal microscope at ×200. Image analysis was performed blind using the Blind Analysis Tool in ImageJ. Images were post-processed using combination of custom ImageJ macros for maximum z-projection, background subtraction (rolling ball radius = 20 microns), brightness and contrast adjustments, thresholding, and particle analysis for semi-automated cell and object counting. All embryonic histology (Nkx2-1, Ki67, Casp3, and St18) was performed following imaging with the Zeiss epifluourescent scope at ×200, and representative images for figures were taken with the Zeiss confocal microscope at ×200. Image analysis was performed blind as with adult tissue. Images were post-processed using a combination of custom ImageJ macros for maximum z-projection, background subtraction (rolling ball radius = 5 microns), brightness and contrast adjustments, thresholding, and particle analysis for semi-automated cell and object counting.

Axonal innvervation was assayed in Ai9 animals by measuring the area of the tdT+ STN signal, delineated as described above, normalized to the total area of the STN measured. The STN was defined in coronal sections between the posterior end of the globus pallidus pars externa (GPe) and the anterior end of the substantia nigra pars reticulata (SNr) dorsal-medial to the cerebral peduncle (cpd), lateral from the lateral hypothalamic area (LHA) and ventral to the zona incerta (ZI) in a clearly delineated area of tdT+ signal.

All Ai34 synaptic puncta image analysis were performed following imaging at ×200 on a Zeiss confocal microscope. Images were post-processed using a combination of custom ImageJ macros for Z-stack slic3e selection, background subtraction (rolling ball radius = 2 microns), brightness and contrast adjustments, thresholding. Synaptic puncta were quantified by particle analysis for semi-automated object counting (ImageJ). All imaging cell counts were normalized to the area of the section being counted.

### MS351 treatment

MS351^61^ [BOC Sciences CAS 472984-79-5]) was reconstituted to a 3 M stock concentration in a solution of 50% water and 50% DMSO (Sigma) per manufacturer specifications. Throughout the entire EB differentiation as well as during the migration assay, or when grown in monolayer cultures, the stock MS351 solution was diluted to 1 mM or we added an equivalent volume of 50% water/50% DMSO as a control. To verify MS351 efficacy on N/D/S cells, MS351 or vehicle-treated day 12 N/D/S EBs were gathered and spun at 300 × *g* to form pellet, washed with 1x PBS, and re-spun at 300 × *g*. We then suspended pellet in Trizol (Invitrogen Cat. 15596026) and lysed the cells with vigorous trituration and incubated the cells for 5 min at room temperature. We then added 200 µL of chloroform (Sigma) and vortexed the lysed cells for 15 s before incubating the cells at room temperature for another 3 min. Next, the cells were spun at 10,000 × *g*, and we removed the clear, aqueous phase from the Trizol reagent and proceeded with RNA mini-prep using the RNeasy Micro Kit (Qiagen Cat. 74004). We next took eluted RNA and proceeded with reverse transcription to create cDNA libraries for RT PCR. Briefly, we add 200 ng of eluted RNA to a mix of water, 1 mM dNTP Solution Mix (New England Biosciences Cat. N0447L), and 5 µM Oligo(dT)20 Primer (Invitrogen Cat. 18418020) and incubated at 65 °C for 5 min. Next, the mixture is cooled on ice for 1 min before adding reagents for reverse transcription from the SuperScript III Reverse Transcriptase Kit (Invitrogen Cat. 18080093) along with 1 unit of RNaseOUT Recombinant RNase Inhibitor (Invitrogen Cat. 10777019) and 1 mM MgCl_2_ (Thermo Fisher Scientific Cat. R0971). This mixture was then incubated at 50 °C for 50 min before being inactivated at 85 °C for 5 min, completing cDNA synthesis. We used the following primer sequences for p16^INK4a^ (also known as Cdkn2A) (Fwd: CAACGACCGAATAGTTACG Rvs: CAGCTCCTCAGCCAGGTC) GAPDH (Fwd: GAGTCCACTGGCGTCTTC Rvs: GGGGTGCTAAGCAGTTGGT) for amplification of 100 ng of cDNA template from N/D/S cells either treated with MS351 or vehicle using Power SYBR Green Master Mix (Thermo Fisher Scientific Cat. 4368577) qRT PCR protocol^62^. qRT PCR reaction was run on a QuantStudio(TM) 7 Flex System in a 96-well format with associated software performing analysis (Applied Biosystems Cat. 4485701).

### RNAscope

e13.5 timed plugs were dissected and immersion fixed with 4% PFA/PBS for 15 min and rinsed with PBS before overnight cryoprotection in 30% sucrose/PBS solution. Following cryoprotection, embryos were mounted in OCT compound and flash frozen on dry ice and stored at −30 °C prior to sectioning. Cryosectioning was performed on a Leica cryostat at 15 µm thickness in the coronal plane, and sections were dried at room temperature for 1 h before storage at −30 °C. The RNAscope assay for fixed-frozen tissue was performed on the sections using the ACD RNAscope Fluorescent Multiplex Reagent Kit (ACD, Cat. 320293) and a catalog probe for St18 transcript (Cat. 443271). Sections were imaged on a Zeiss confocal microscope at ×200.

### MGE explant migration assay

Nkx2.1^Cre^, Ai9 double homozygote male studs were crossed to female breeders to generate timed pregnancies, day of vaginal plug was considered e0.5. Heterozygous embryos were collected at e12.5, TdT fluorescence confirmed, and MGEs were dissected in ice-cold PBS and placed into the center of a black 96-well optical bottom plastic tissue culture plates (Ibidi). Excess PBS was gently removed before well and MGE explants were coated with 200ul of Matrigel (Corning) droplets diluted 1:1 in Neurobasal (NB) media (Gibco 12348-017) supplemented with B27 and penstrep antibiotic (NB/B27 media) using pre-chilled P1000 pipette tips. Explants were incubated for 30 min at 37 °C. Wells were then covered with NB/B27 media (with or without MS357 (BOC Sciences 472984-79-5) at 1 nM concentration) and cultured with daily media changes for 4 days until fixation in 4% PFA in PBS for 10 min. Explants were stained with DAPI (1:1000) for 5 min and coated with Aqua-Poly/Mount (Polysciences 18606). Entire well was imaged at ×40 magnification on a Zeiss Apotome 2 using Zen Blue software.

### Migration assay quantification

Individual tiles of CZI tiled images (300 tiles/image) were batch exported as OME TIFFs and z stacks were then merged into a single, focused composite image using the Extended depth of field ImageJ plugin^105^. Resulting images were then converted to h5 format for pixel classification using ilastik v1.3.3^106^. Ilastik pixel classifier was trained to distinguish TdT/DAPI double-positive nuclei from background on 30 selected tile images encompassing a range explant image regions with high accuracy. After training, all prepared images from explant wells were batch analyzed using ilastik pixel classifier and predictions were exported as simple segmented image. Segmented prediction files were re-stitched based on tiling information extracted from initial czi file using ImageJ stitching plugin^107^ and overlayed with original image to check for accuracy. Locations of ilastik predicted nuclei in the stitched segmented images were then identified using ImageJ analyze particles functionality, with a size threshold of 5 to reduce non-cellular particles. X,Y (X1,Y1) coordinates of identified nuclei were then exported and their distance from explant edge was calculated by a simple Euclidian distance formula (SQRT((X1−X2)^2 ^+ (Y1−Y2)^2^)) from center of explant minus the radius of the explant. X,Y coordinate of the explant center (X2,Y2) and perimeter were calculated using ImageJ trace tool. Five explants were generated per condition, some were dislodged during the protocol, leaving 3 control explants and 4 treated explants. 25,962 and 33,403 total objects counted from control and treated explants with an average of 8654 and 8350 objects per explant, respectively, in each condition. Distance migrated was plotted using GraphPad prism and Kolmogorov–Smirnov test performed for statistical significance.

### Reporting summary

Further information on research design is available in the Nature Portfolio Reporting Summary linked to this article.

## Supplementary information

Supplementary Information

Reporting Summary

## Data Availability

In addition, scRNA-seq and bulk RNA-seq datasets are available from NCBI GEO under Accession Numbers GSE181349 and GSE180825. Source data are provided with this paper.

## Source data

Source Data
