# Supplementary material for: St18 specifies globus pallidus projection neuron identity in MGE lineage
Source: Nat Commun. 2022 Dec 14;13:7735. doi: 10.1038/s41467-022-35518-5 (PMC9751150; doi:10.1038/s41467-022-35518-5)
Supplement: Supplementary file 1 — Supplementary Information [file 41467_2022_35518_MOESM1_ESM.pdf]

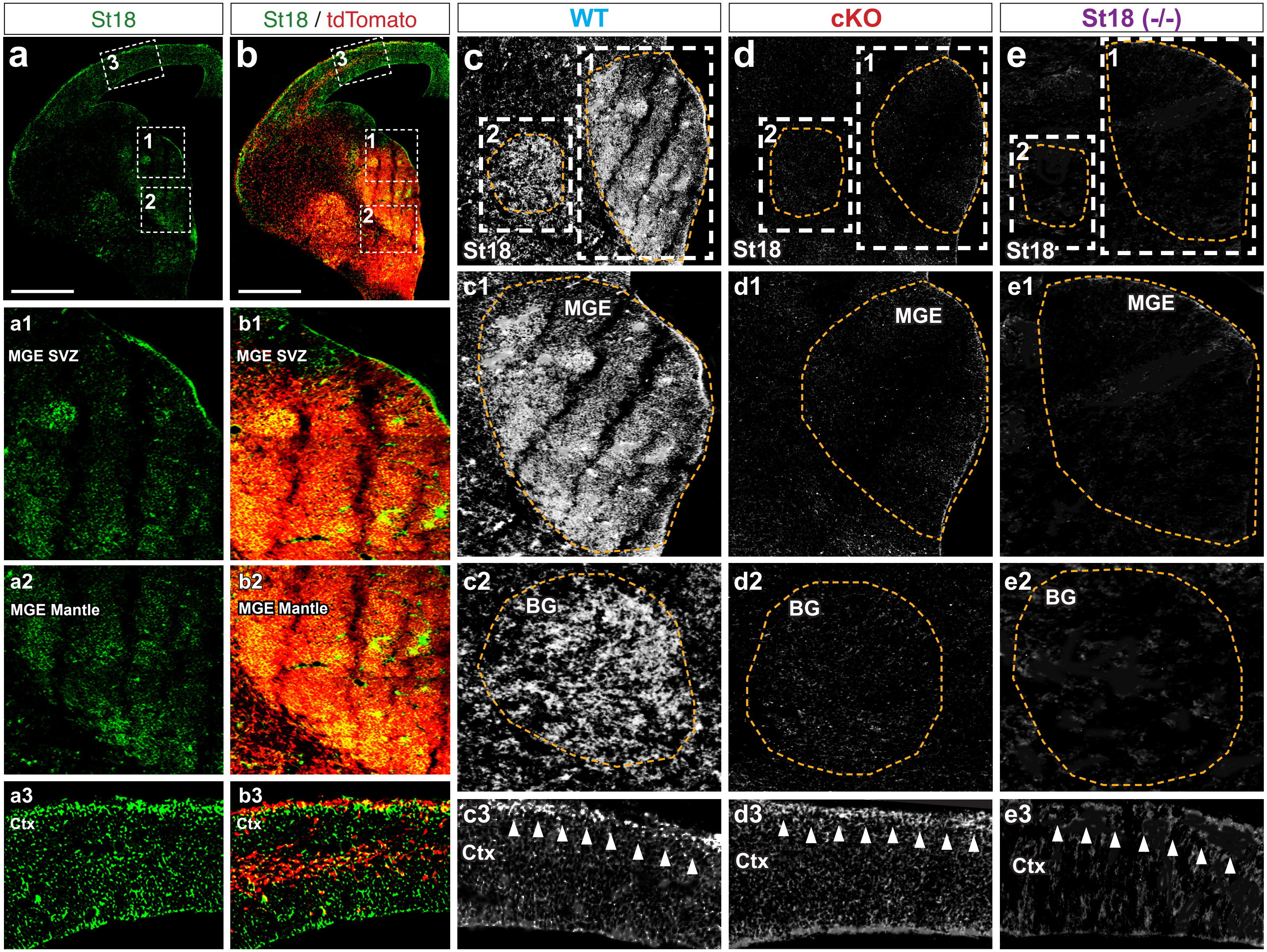

Figure S1. ***Validation of St18 antibody in WT, St18 cKO, and St18 (-/-).*** E13.5 embryonic MGE. (a) WT E13.5 MGE immunolabelled with St18 antibody. Insets indicate MGE (1) and GP (2). CP (3) shown below with arrowheads indicating location of St18 labelling. (b) St18 cKO E13.5 MGE immunolabelled with St18. Subsets are the same as (a). (c) St18 (-/-) E13.5 MGE immunolabelled with St18. Subsets are the same as (A). Immunohistochemistry repeated across 3 biological replicates. Scale bar represents 500 microns.

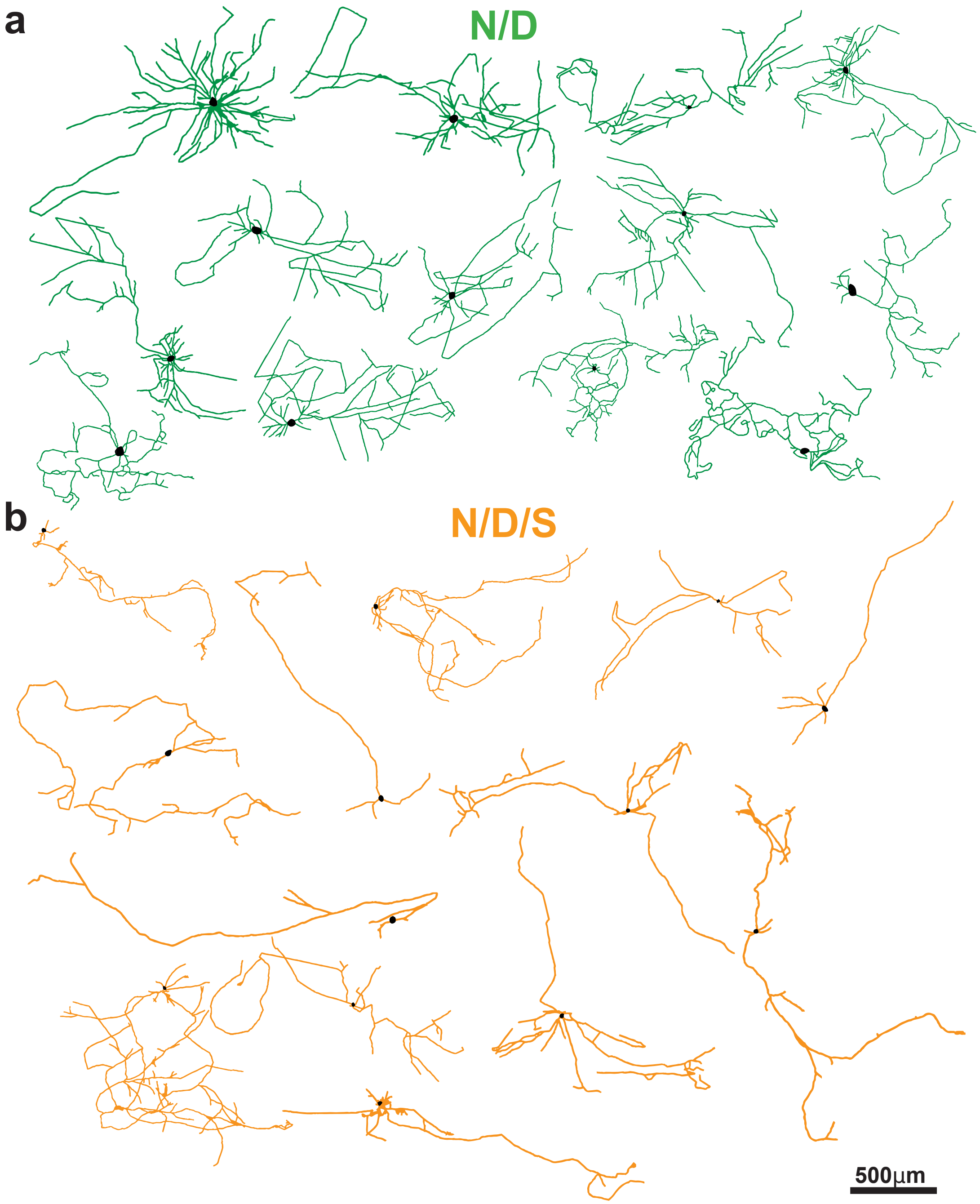

Figure S2. ***Additional morphological reconstructions of N/D and N/D/S neurons.*** Additional 2D tracings of ES-derived MGE neurons cultured in isolation on unlabeled cortical feeders. (a) N/D neurons in green. (b) N/D/S neurons in orange.

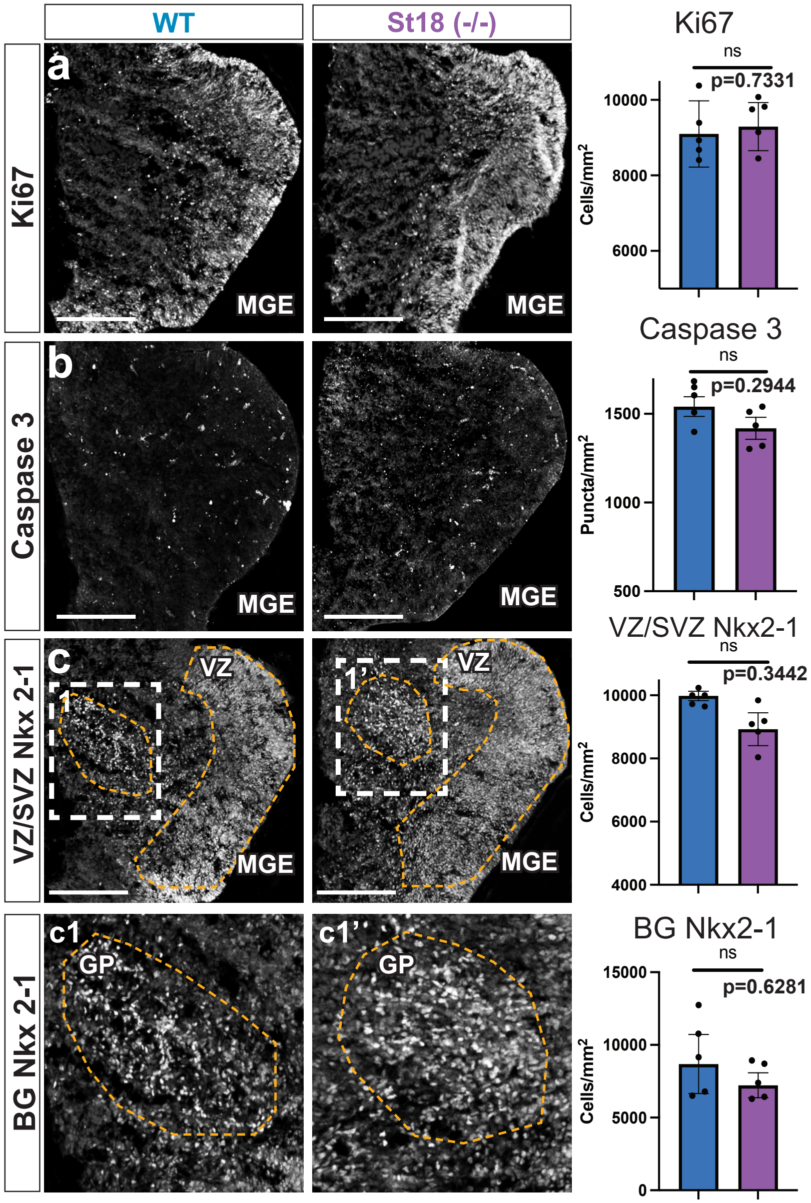

Figure S3. ***Whole animal St18 KO does not affect MGE proliferation, cell-death, nor MGE lineage specification at E13.5.*** (a) WT and St18 (-/-) E13.5 MGE with immunolabelled Ki67 (Unpaired t-test). (b) WT and St18 (-/-) E13.5 MGE with immunolabelled Casp3 (Unpaired t-test). (c) WT and St18 (-/-) E13.5 MGE with immunolabelled Nkx2-1. VZ is outlined in orange. Inset shows higher-powered imaged BG (Unpaired t-test). All datasets represent N=5 per genotype. Data are presented as mean +/- SEM. Source data are provided as a Source Data file. Scale bar represents 250 microns.

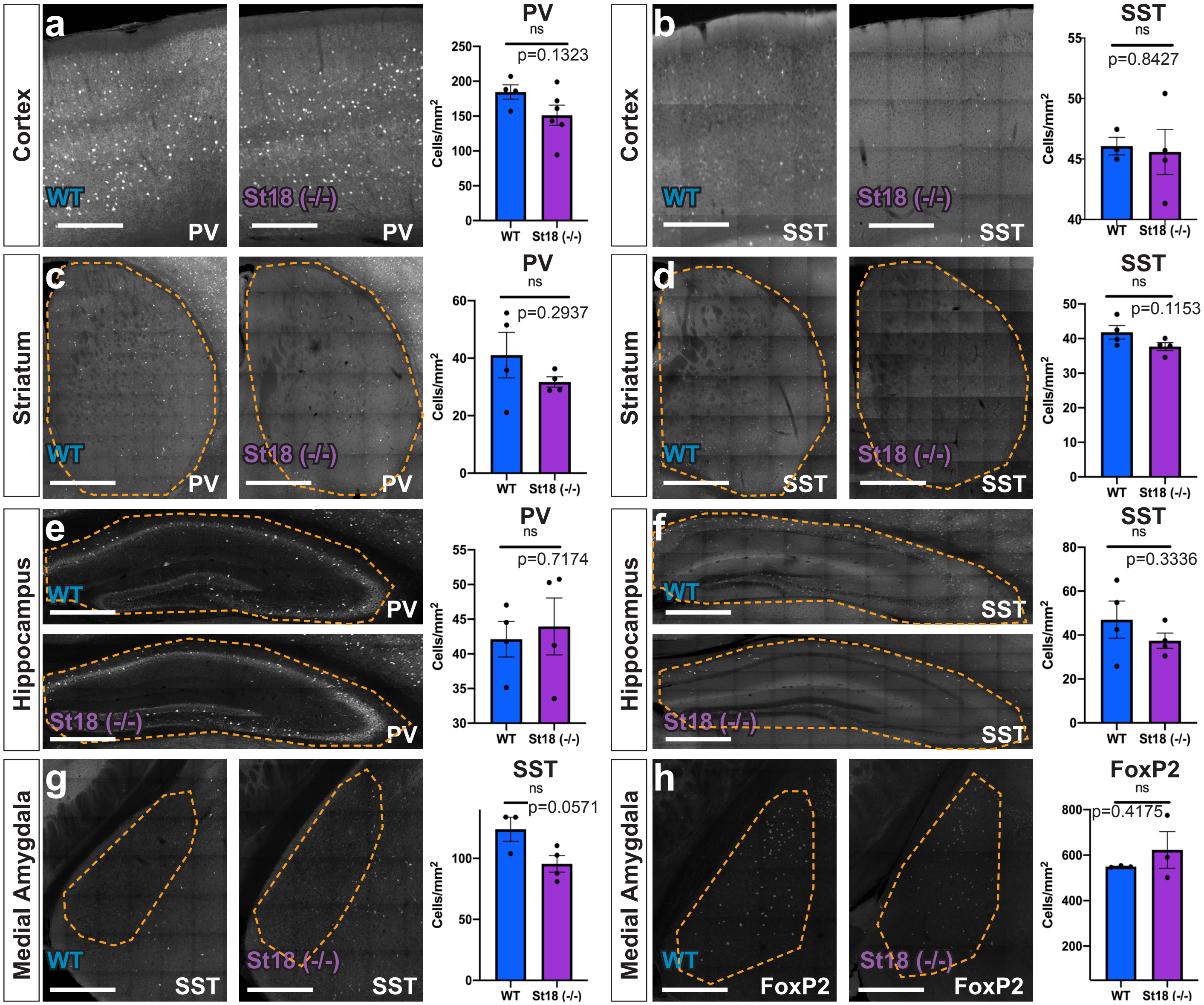

Figure S4. ***Whole animal St18 KO does not affect MGE lineages of the cortex, striatum, hippocampus, and MeA.*** Quantification of (a) PV+ (N= 4 WT and 6 St18 (-/-)) and (b) SST+ (N= 3 WT and 4 St18 (-/-)) neurons in the cortex. Quantification of (c) PV+ (N= 4 WT and 4 St18 (-/-)) and (d) SST+ (N= 4 WT and 4 St18 (-/-)) neurons in the striatum. Quantification of (e) PV+ (N= 4 WT and 4 St18 (-/-)) and (f) SST+ (N= 4 WT and 4 St18 (-/-)) neurons in the hippocampus. Quantification of (g) SST+ (N= 3 WT and 4 St18 (-/-)) and (h) FoxP2+ (N= 3 WT and St18 (-/-)) neurons in the MeA. All data assessed by Unpaired t-test. Data are presented as mean +/- SEM. Source data are provided as a Source Data file.

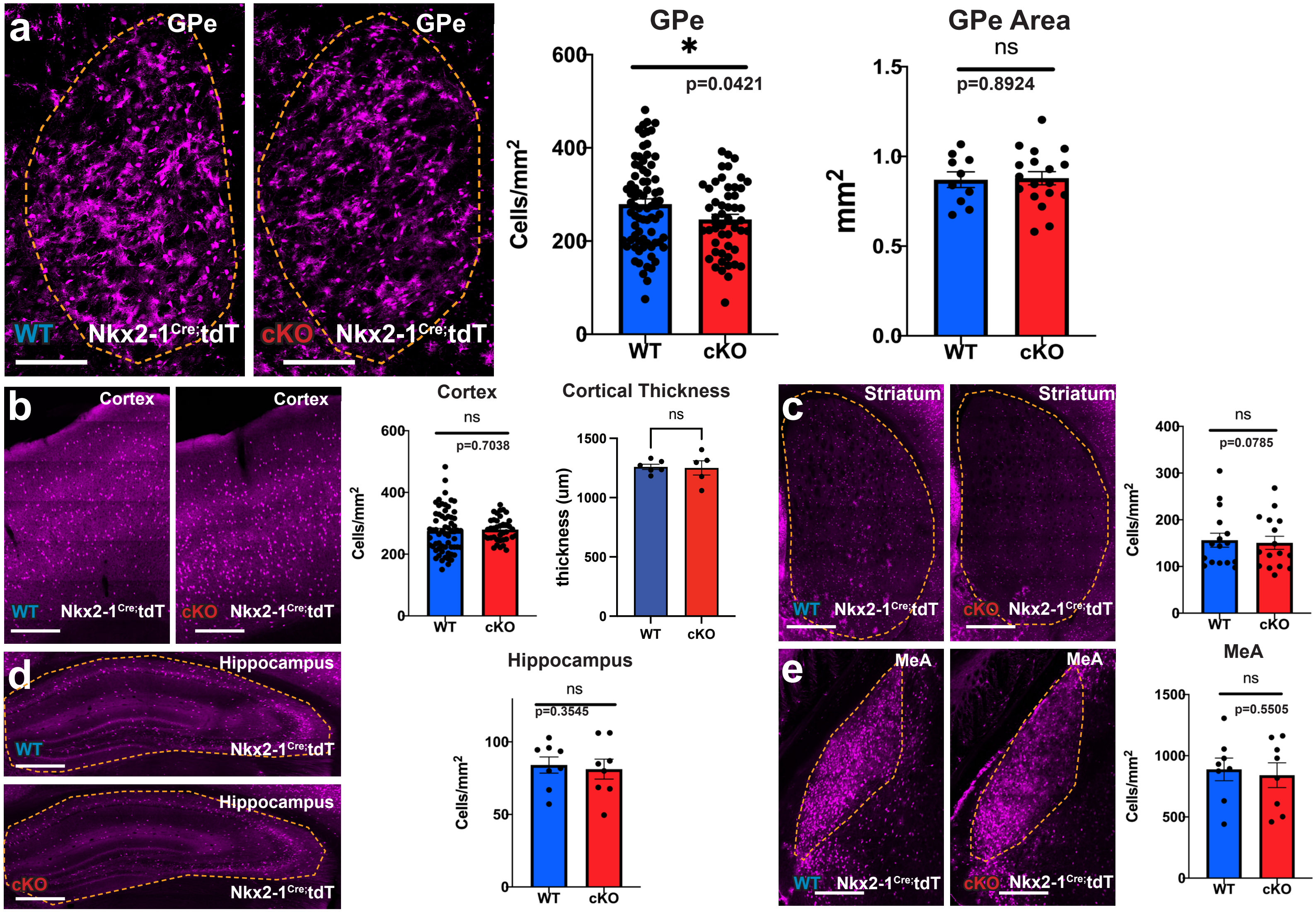

Figure S5. ***St18 conditional ablation in the MGE produces a specific loss of fate-mapped tdTomato+ neurons in the GPe.*** (a) WT and St18 cKO GPe with MGE lineage fate-map (Nkx2-1Cre; Ai9) (Unpaired t-test; N= 76 WT and 50 St18 cKO). (b) WT and St18 cKO Cortex with MGE lineage fate-map (Nkx2-1Cre; Ai9) (Unpaired t-test; N= 63 WT and 38 St18 cKO). (c) WT and St18 cKO Hippocampus with MGE lineage fate-map (Nkx2-1Cre; Ai9) (Unpaired t-test; N= 8 WT and 8 St18 cKO). (d) WT and St18 cKO MeA with MGE lineage fate-map (Nkx2-1Cre; Ai9) (Unpaired t-test; N= 8 WT and 8 St18 cKO). (e) WT and St18 cKO Striatum with MGE lineage fate-map (Nkx2-1Cre; Ai9) (Unpaired t-test; N= 16 WT and 16 St18 cKO). Data are presented as mean +/- SEM. Source data are provided as a Source Data file. Scalebar represents 500 microns.

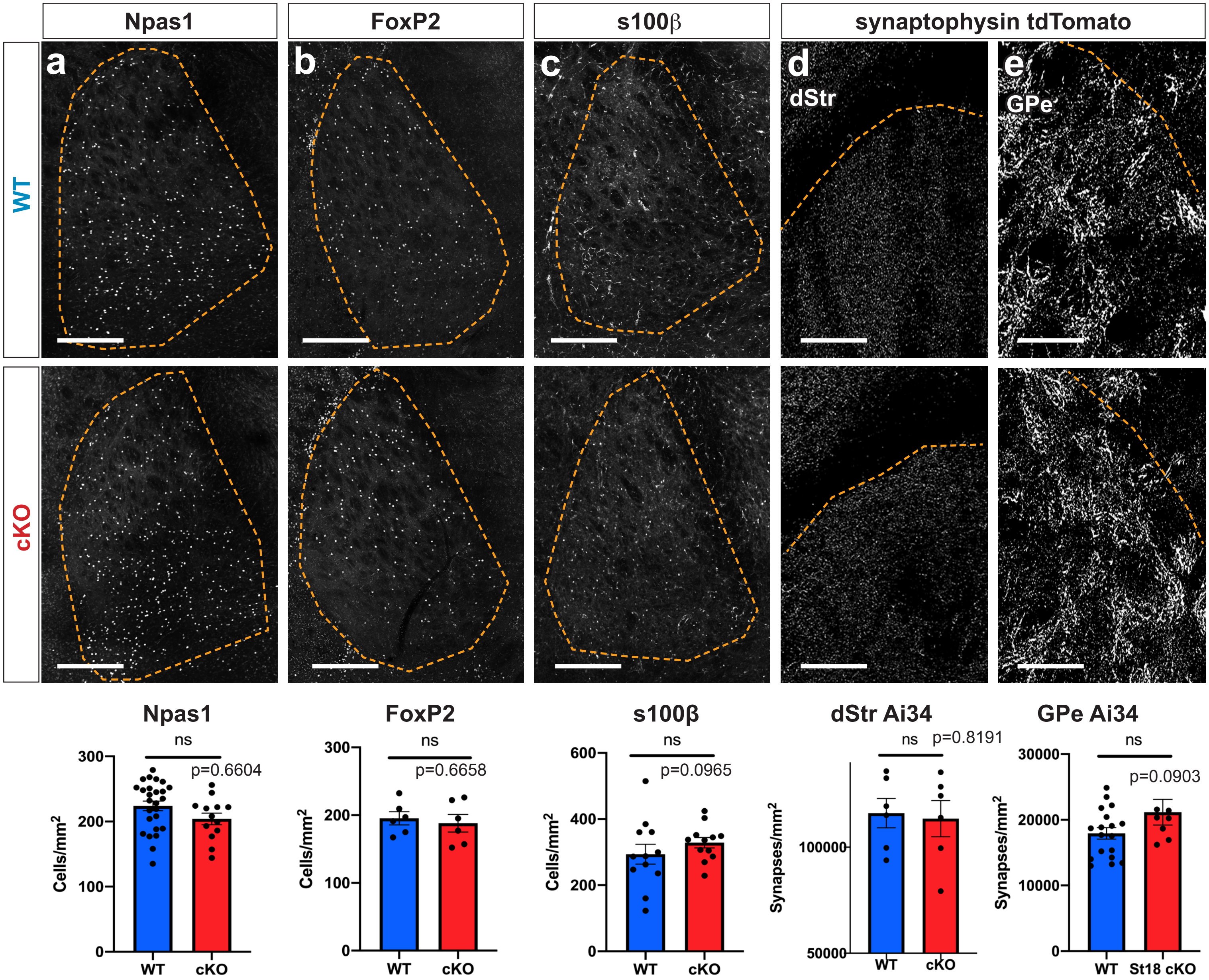

Figure S6. ***St18 cKO does not cause any change in the arkypallidal neuron population in the GPe.*** (a) Quantification of Npas1+ neurons (Unpaired t-test; N= 26 WT and 13 St18 cKO). (b) Quantification of FoxP2+ neurons (Unpaired t-test; N= 6 WT and 6 St18 cKO). (c) Quantification of s100b glia cells (Unpaired t-test; N= 12 WT and 12 St18 cKO). (d) Quantification of Ai34 synaptic puncta density in the dStr (Unpaired t-test; N= 6 WT and 6 St18 cKO). (e) Quantification of Ai34 synaptic puncta density in the GPe (Unpaired t-test; N= 18 WT and 8 St18 cKO). Data are presented as mean +/- SEM. Source data are provided as a Source Data file. Scalebar represents 500 microns.

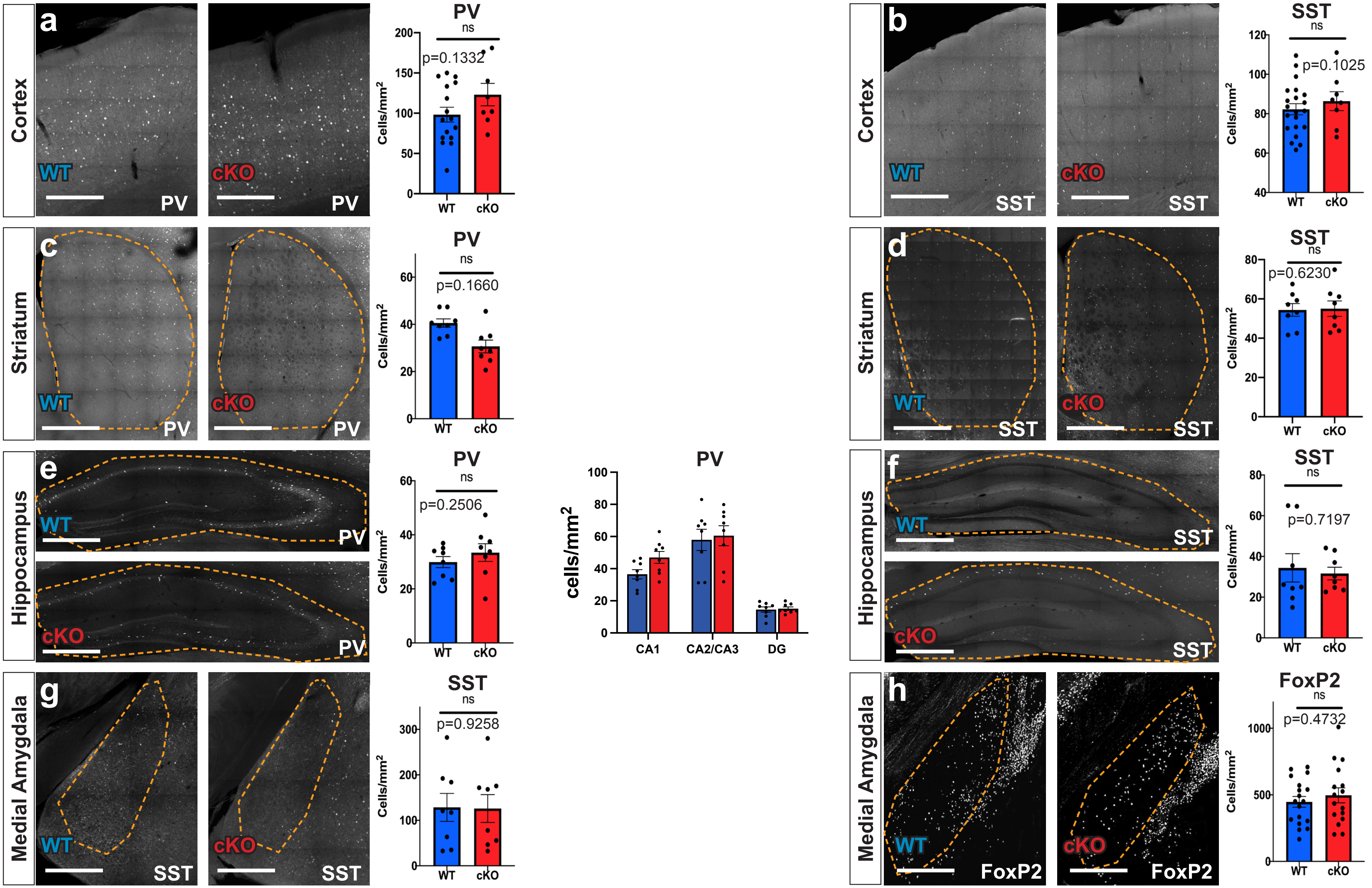

Figure S7. ***Conditional St18 KO does not affect MGE lineages of the cortex, striatum, hippocampus, and MeA.*** Quantification of (a) PV+ (N= 16 WT and 8 St18 cKO) and (b) SST+ (N= 21 WT and 8 St18 cKO) neurons in the cortex. Quantification of (c) PV+ (N= 8 WT and 8 St18 cKO) and (d) SST+ (N= 8 WT and 8 St18 cKO) neurons in the Striatum. Quantification of (e) PV+ (N= 8 WT and 8 St18 cKO) in the hippocampus as a whole or by region (CA1 vs. CA2/3 vs. DG). (f) SST+ (N= 8 WT and 8 St18 cKO) neurons in the hippocampus. Quantification of (g) SST+ (N= 8 WT and 8 St18 cKO) and (h) FoxP2+ (N= 18 WT and 16 St18 cKO) neurons in the MeA. All data assessed by Unpaired t-test. Data are presented as mean +/- SEM. Source data are provided as a Source Data file. Scalebar represents 500 microns.

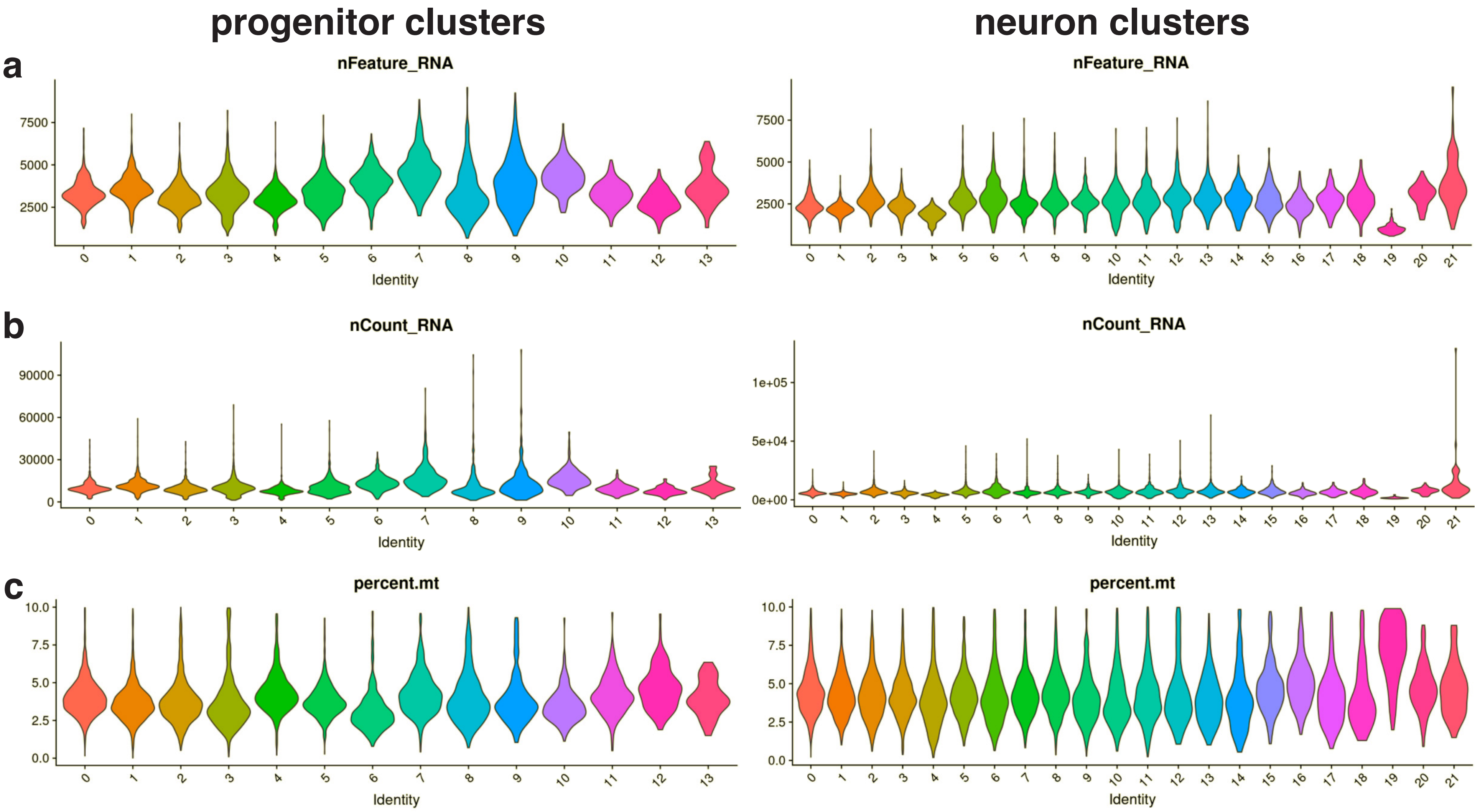

Figure S8. ***Quality control of single cell RNA-seq dataset.*** Violin plots showing the distribution of (a) unique genes detected per cell, (b) Unique molecular identifier (UMI) counts per cell, and (c) percent of UMIs mapped to the mitochondrial genome, grouped by cluster. Left, progenitor clusters; right, neuron clusters. Cells with low unique genes detected or high percentage of mitochondrial reads were removed prior to clustering.

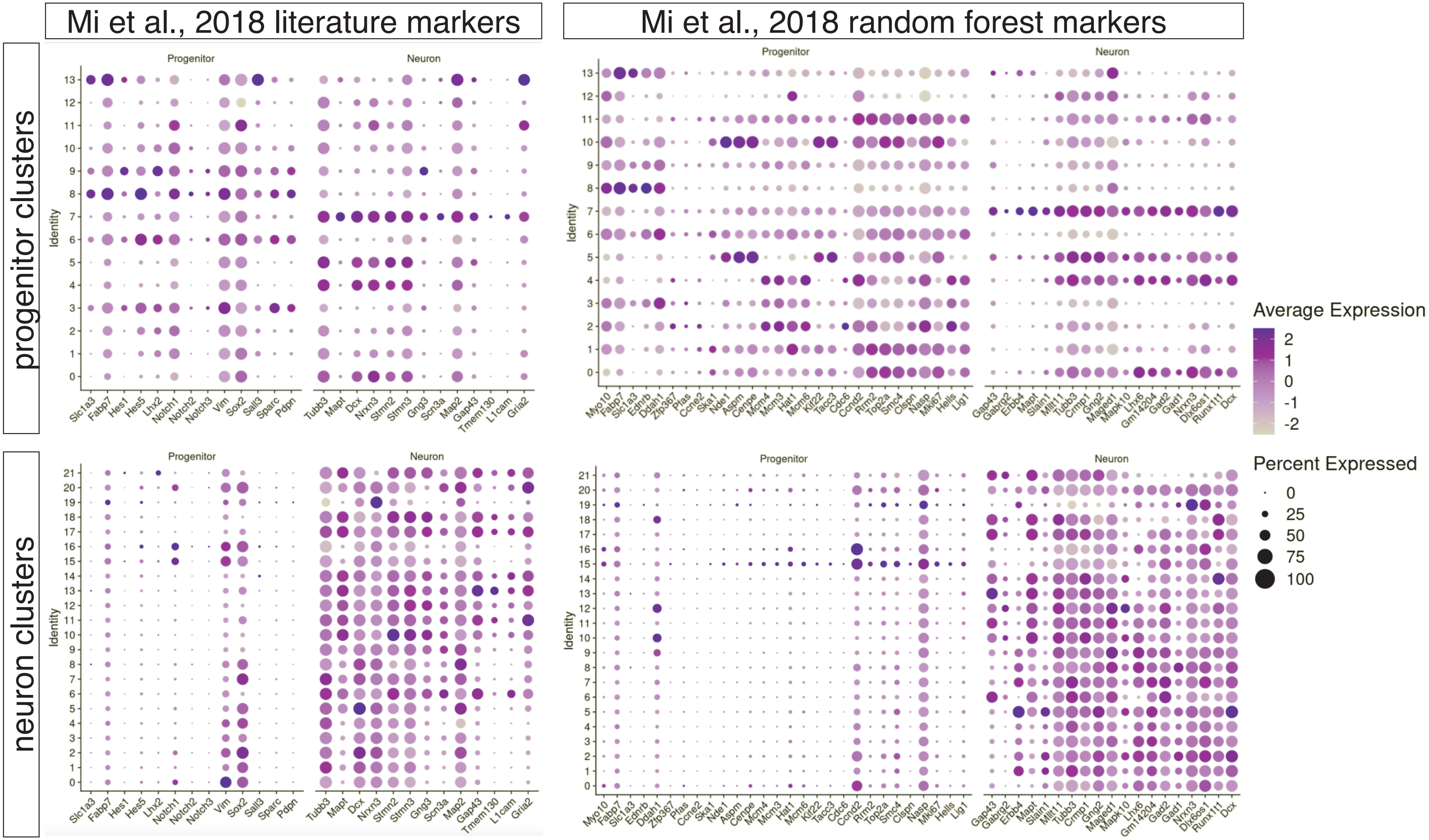

Figure S9. ***Classification of neuronal and progenitor clusters.*** Dot plots showing scaled average expression of genes from (Mi et al. 2018) distinguishing neuron and progenitor clusters. Left column, established neuron and progenitor markers from literature. Right column, neuron and progenitor markers as selected by a random forest classifier. Top row, expression of clusters classified as progenitor. Bottom row, expression of clusters classified as neuronal. Clusters were assigned to the class whose markers show the greatest average expression.

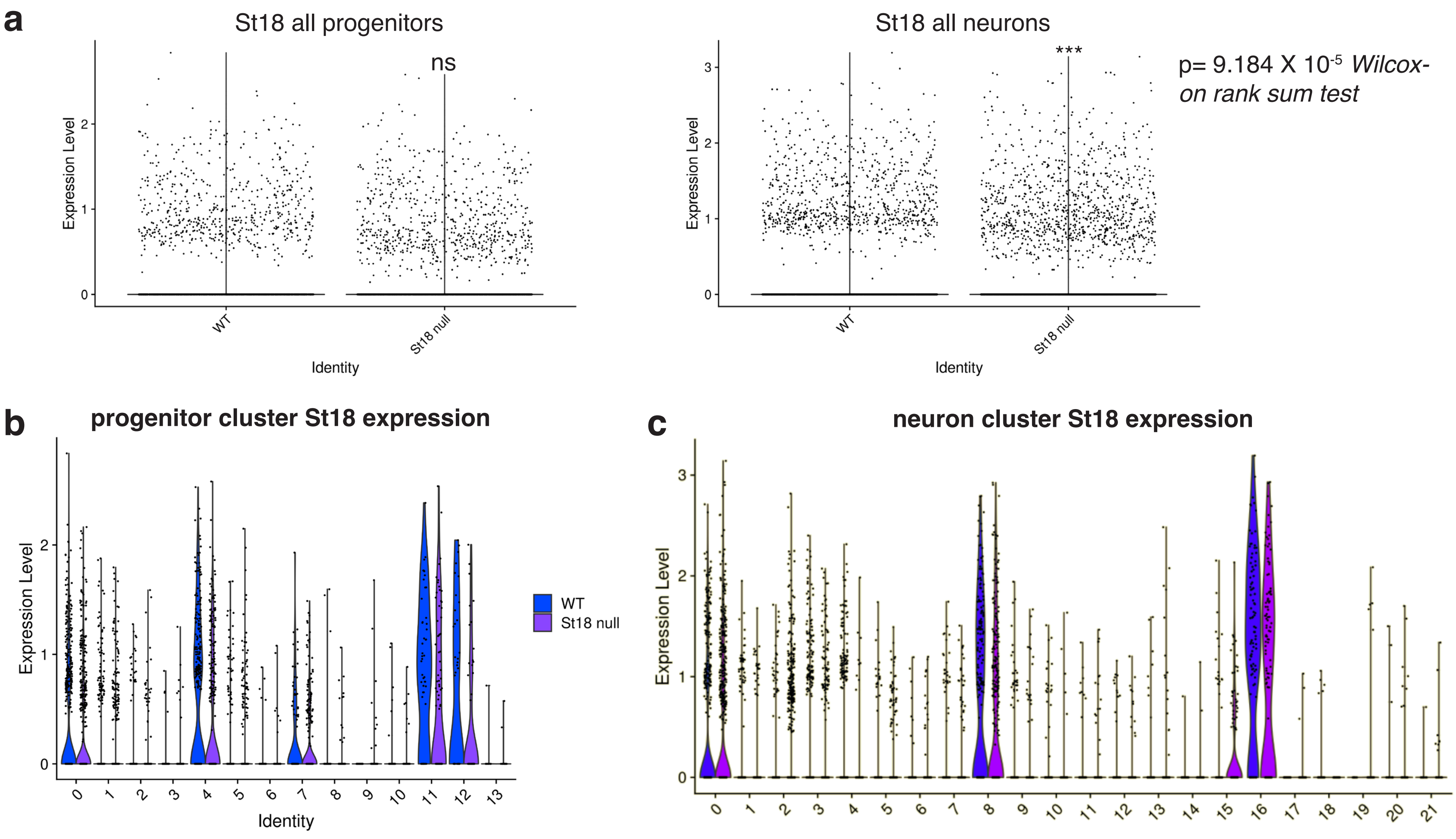

Figure S10. ***Expression of St18 in MGE single cell RNA-seq.*** (a) Violin plot showing log-normalized expression of St18 in WT and St18 null progenitors (left) and neurons (right). (b,c) Violin plots of log-normalized expression in WT and St18 null (b) progenitors or (c) neurons, grouped by cluster.

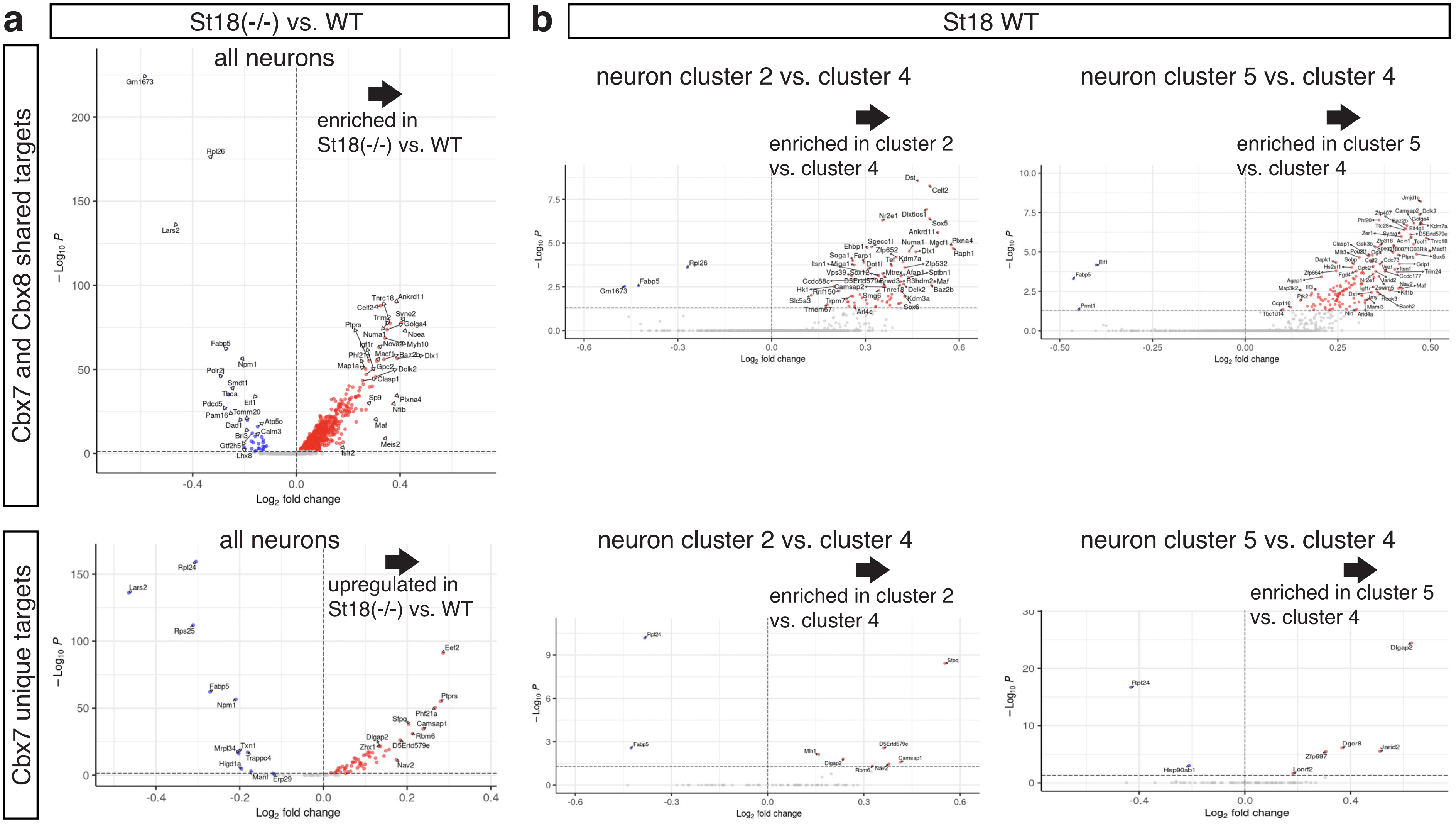

Figure S11. ***Gene expression of identified Cbx 7/8 shared targets across St18 genotypes and neuronal clusters.*** Cbx7 and Cbx8 shared targets and gene targets unique to Cbx7 were identified in a previous study (Klauke et al., 2013). (a) MA plots comparing St18(-/-) and WT datasets for all neurons. Top, Cbx7 and Cbx8 shared target gene targets (581 targets enriched in St18(-/-); 35 depleted); Bottom, Cbx7 unique target genes (53 targets enriched in St18(-/-); 11 depleted). (b) MA plots comparing neuron cluster 4 vs. 2 (left; 77 targets enriched in 2; 2 depleted) and 5 (right; 151 targets enriched in 5; 3 depleted). Top row shows Cbx7 and Cbx8 shared targets, bottom row shows Cbx7 unique targets.

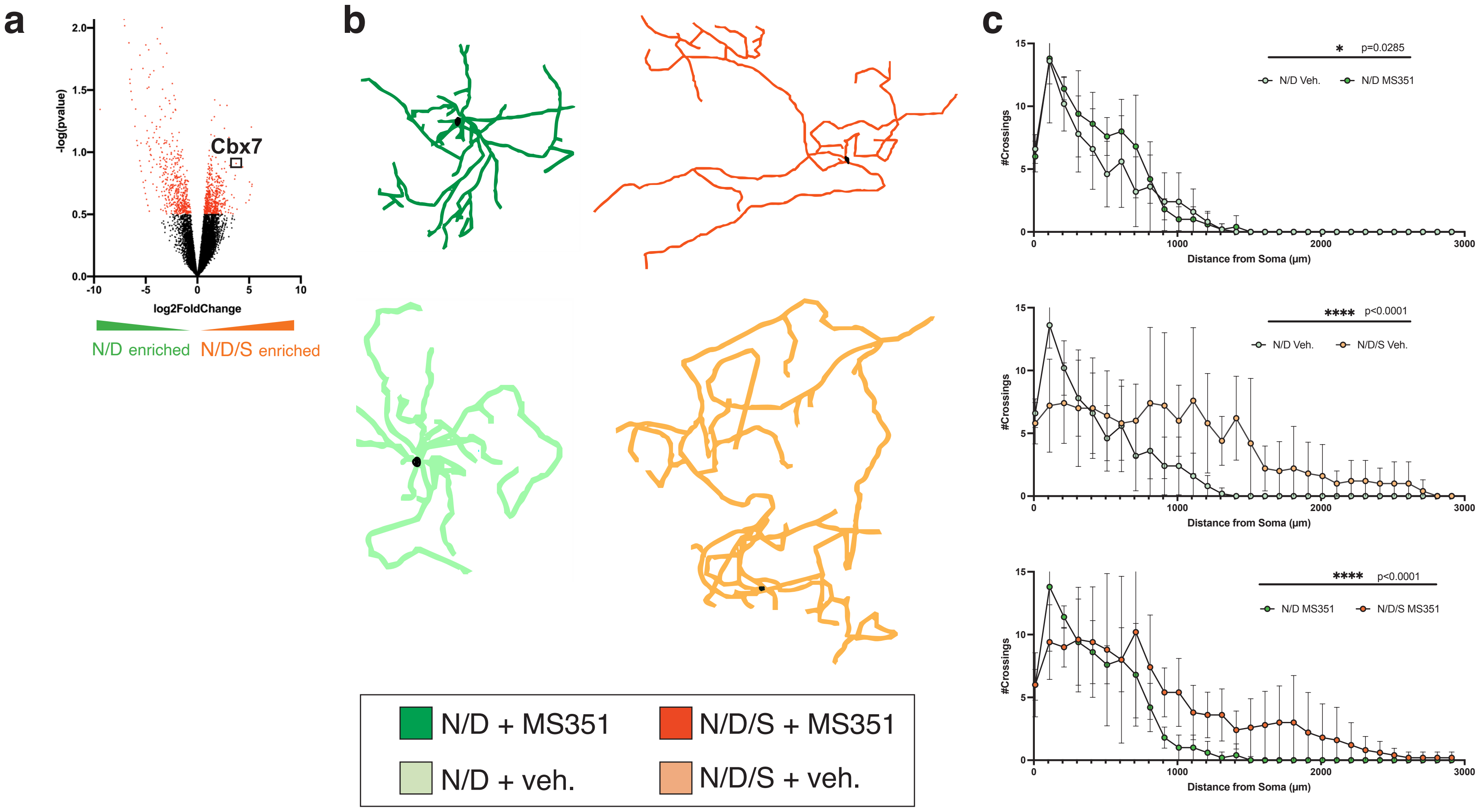

Figure S12. MS351 administration does not affect morphology of N/D and N/D/S neurons. (a) MA plot of differentially expressed genes in N/D/S neurons compared to N/D neurons; Cbx7 is highlighted. (b) Representative morphological reconstructions of N/D neurons and N/D/S neurons treated with MS351 or DMSO vehicle. (c) Sholl analysis of (top) N/D vehicle vs. N/D MS351; (middle; $p=0.000000264$) N/D vehicle vs. N/D/S vehicle; (bottom; $p= 0.0000501$) N/D MS351 vs. N/D/S MS351. Scholl analyzed by 2-way ANOVA, multiplicity-adjusted p-value. N= 10 N/D/S MS351 and 10 N/D/S Veh. and 10 N/D MS351 and 10 N/D Veh. Data are presented as mean +/- SEM. Source data are provided as a Source Data file.
