# Supplementary material for: St18 specifies globus pallidus projection neuron identity in MGE lineage
Source: Nat Commun. 2022 Dec 14;13:7735. doi: 10.1038/s41467-022-35518-5 (PMC9751150; doi:10.1038/s41467-022-35518-5)
Supplement: Supplementary file 2 — Reporting Summary [file 41467_2022_35518_MOESM2_ESM.pdf]

nature portfolio

Corresponding author(s): Edmund Au, Ph.D.

Last updated by author(s): Dec 5, 2022

Reporting Summary

Nature Portfolio wishes to improve the reproducibility of the work that we publish. This form provides structure for consistency and transparency in reporting. For further information on Nature Portfolio policies, see our Editorial Policies and the Editorial Policy Checklist.

Statistics

For all statistical analyses, confirm that the following items are present in the figure legend, table legend, main text, or Methods section.

n/a	Confirmed	
☐	☒	The exact sample size (*n*) for each experimental group/condition, given as a discrete number and unit of measurement
☐	☒	A statement on whether measurements were taken from distinct samples or whether the same sample was measured repeatedly
☐	☒	The statistical test(s) used AND whether they are one- or two-sided<br>*Only common tests should be described solely by name; describe more complex techniques in the Methods section.*
☐	☒	A description of all covariates tested
☐	☒	A description of any assumptions or corrections, such as tests of normality and adjustment for multiple comparisons
☐	☒	A full description of the statistical parameters including central tendency (e.g. means) or other basic estimates (e.g. regression coefficient) AND variation (e.g. standard deviation) or associated estimates of uncertainty (e.g. confidence intervals)
☐	☒	For null hypothesis testing, the test statistic (e.g. *F*, *t*, *r*) with confidence intervals, effect sizes, degrees of freedom and *P* value noted<br>*Give P values as exact values whenever suitable.*
☒	☐	For Bayesian analysis, information on the choice of priors and Markov chain Monte Carlo settings
☒	☐	For hierarchical and complex designs, identification of the appropriate level for tests and full reporting of outcomes
☒	☐	Estimates of effect sizes (e.g. Cohen's *d*, Pearson's *r*), indicating how they were calculated

Our web collection on statistics for biologists contains articles on many of the points above.

Software and code

Policy information about availability of computer code

Data collection	Note: no custom code was used to collect data. Below is a summary of software used for data collection.<br><br>Images were obtained on Zeiss Microscopy native software, Zen.<br><br>bulk RNA-seq: RNA was purified using an RNA miniprep kit (Zymo) and measured with a Bioanalyzer (Agilent) to ensure an RIN score of7 or greater. mRNA was enriched using a standard poly-A pull-down prior to library construction with lllumina TruSeq chemistry. The library is sequenced using lllumina NovaSeq 6000 at the Columbia Genome Center. Samples were multiplexed in each lane with yields of paired-end l00bp reads per sample.<br><br>scRNA-seq: Samples were submitted to the Sulzberger Genome Center Single Cell Core at the Columbia University Medical Center for library construction using l0X Genomics chemistry. Sequencing is run on a l0X Genomics platform, and analysis was performed on l0X Genomics' Cellranger software version 5.0.1.
Data analysis	Note: no custom code was used to analyze data. Below is a summary of software used for data analysis.<br>Images were processed by ImageJ (2.9.0) maximum z-projection, background subtraction (rolling ball radius=S-20 microns, depending on image), brightness and contrast adjustments, thresholding, and particle analysis for semi-automated cell and object counting.<br><br>Bulk RNA seq data: RTA (lllumina) was used for base calling and bcl2fastq2 (version 2.20) for fastq format conversion. A pseudoalignment was performed to a kallisto index created from the mouse transcriptome using kallisto (0.44.0). We tested for differentially expressed genes using Sleuth (0.30.1), an R package designed for differential gene analysis from kallisto abundance files.

scRNA-seq: cells were filtered based on the following criteria: cells with fewer than 200 unique genes detected, or cells with more than 10% of reads mapping to the mitochondrial genome were excluded from further analysis. After QC, all cells from all experiments were aggregated and clustered using the Seurat (4.1.0) package in R, with default parameters, except the following: cell cycle differences among dividing cells were regressed and removed (Mi et al., 2018), and clustering was performed on the 49 principal components with a p-value less than 0.01, as determined by jackstraw resamplingl0, with a resolution of 1.4, based on bootstrapped iterative clusteringll. After clustering, progenitor and neuronal clusters were identified by plotting the expression of marker genes from12. Subsequently, progenitors and neurons were re-clustered separately. Each cluster was then assessed for statistically significant enrichment or depletion in the WT versus St18 (-/-) groups, using a Fisher's exact test with Bonferroni correction for multiple comparisons. This identified three neuronal clusters with statistically significant differences between the groups in terms of proportions, with the additional constraints of comprising >5% of the total neuronal population and at least two-fold change in proportions between the groups. Differential gene expression among the clusters was performed using a Wilcoxon rank-sum test in the Seurat package.

llastik (1.3.3) (Berg et al., 2019) was used to derive image channels for analysis of MGE explants. After user training, llastik employed pixel classification in order to generate images that were suitable for downstream segmentation by FIJI. Segmented objects were then mapped onto stitched file to determine migratory distances for Nkx2-1Cre-fatemapped MGE neurons.

For manuscripts utilizing custom algorithms or software that are central to the research but not yet described in published literature, software must be made available to editors and reviewers. We strongly encourage code deposition in a community repository (e.g. GitHub). See the Nature Portfolio guidelines for submitting code & software for further information.

Data

Policy information about availability of data

All manuscripts must include a data availability statement. This statement should provide the following information, where applicable:

- Accession codes, unique identifiers, or web links for publicly available datasets
- A description of any restrictions on data availability
- For clinical datasets or third party data, please ensure that the statement adheres to our policy

Source data are provided with this paper as a separate file Source Data.xls. Additionally, scRNA-seq and bulk RNA-seq datasets are available from NCBI GEO (Accession Numbers: GSE181349 and GSE180825).

Human research participants

Policy information about studies involving human research participants and Sex and Gender in Research.

Reporting on sex and gender

Use the terms sex (biological attribute) and gender (shaped by social and cultural circumstances) carefully in order to avoid confusing both terms. Indicate if findings apply to only one sex or gender; describe whether sex and gender were considered in study design whether sex and/or gender was determined based on self-reporting or assigned and methods used. Provide in the source data disaggregated sex and gender data where this information has been collected, and consent has been obtained for sharing of individual-level data; provide overall numbers in this Reporting Summary. Please state if this information has not been collected. Report sex- and gender-based analyses where performed, justify reasons for lack of sex- and gender-based analysis.

Population characteristics

Describe the covariate-relevant population characteristics of the human research participants (e.g. age, genotypic information, past and current diagnosis and treatment categories). If you filled out the behavioural & social sciences study design questions and have nothing to add here, write "See above."

Recruitment

Describe how participants were recruited. Outline any potential self-selection bias or other biases that may be present and how these are likely to impact results.

Ethics oversight

Identify the organization(s) that approved the study protocol.

Note that full information on the approval of the study protocol must also be provided in the manuscript.

Field-specific reporting

Please select the one below that is the best fit for your research. If you are not sure, read the appropriate sections before making your selection.

☒ Life sciences ☐ Behavioural & social sciences ☐ Ecological, evolutionary & environmental sciences

For a reference copy of the document with all sections, see nature.com/documents/nr-reporting-summary-flat.pdf

Life sciences study design

All studies must disclose on these points even when the disclosure is negative.

Sample size

Power analysis was performed (Matlab sampsizepwr, beta=0.2, power=0.8) and it was determined that 6 biological replicates were needed for St18 mutant vs. wildtype control. Similar analysis was performed for in vitro work and found minimum 4 biological replicates.

Data exclusions	No data was excluded.
Replication	Data was reproducible across replicates. Replicate numbers listed in Figure Legends.
Randomization	mutant and wildtypes were obtained as littermates and analyzed until the requisite number of biological replicates were obtained and often exceeded.
Blinding	All data was image data blinded using ImageJ Blind Analysis Tool prior to analysis and unblinded afterwards.

Reporting for specific materials, systems and methods

We require information from authors about some types of materials, experimental systems and methods used in many studies. Here, indicate whether each material, system or method listed is relevant to your study. If you are not sure if a list item applies to your research, read the appropriate section before selecting a response.

Materials & experimental systems

n/a	Involved in the study
☐	☒ Antibodies
☐	☒ Eukaryotic cell lines
☒	☐ Palaeontology and archaeology
☐	☒ Animals and other organisms
☒	☐ Clinical data
☒	☐ Dual use research of concern

Methods

n/a	Involved in the study
☒	☐ ChIP-seq
☒	☐ Flow cytometry
☒	☐ MRI-based neuroimaging

Antibodies

Antibodies used	PV [ImmunoStar Cat. 24428] 1:1000,<br>PV [Swant Cat. 235] 1:1000<br>Npasl [Gift antibody generated in Chan Lab (not commercially available), Northwestern University] 1:1000, FoxP2 [Gift antibody generated in Jessel Lab (not commercially available), Columbia University] 1:1000, Er81 [Gift antibody generated in Jessel Lab (not commercially available), Columbia University] 1:32000,<br>SST [Millipore Sigma Cat. MAB354] 1:250,<br>sl00β [Millipore Sigma Cat. SAB5600115] 1:1000, Nkx2-1 [Abeam Cat. Ab227652] 1:500,<br>Ki67 [Invitrogen Cat. 14-5698-82] 1:200, Caspase 3 [Novus Cat. 31A1067] 1:500<br>Rat-anti St18 antibody was produced as follows: N-terminus of St18 (aa 60-298) was fused to a maltose binding protein to generate a purified immunogen. Strategic Bio-Solutions (Newark, DE) used the immunogen to generate rat polyclonal anti-St18 antibody.z¬¬¬¬
Validation	St18 antibody was validated by testing for signal on WT vs. St18 (-/-) vs. St18 cKO (Nkx2.1-Cre) embryonic tissue. Here, we noted St18 signal in cortex and MGE in WT, absence of St18 signal in St18 (-/-) and absence of S18 signal in MGE in St18 cKO. See Supplemental Figure 1 panels c -e.

Eukaryotic cell lines

Policy information about cell lines and Sex and Gender in Research

Cell line source(s)	We derived a mouse parental ES cell line from a Dlx6aCre (Jackson Laboratory Stock No. 008199); Ai9 (Jackson Laboratory Stock No. 007909) e3.5 pre-implantation male embryo using previously described methods (Meissner et al., 2009 Methods in Molecular Biology)
Authentication	Authentication performed by directed differentiation of ES line and validation of tdTomato+ neurons (McKenzie et al., 2019 Neuron) and by RNA-seq in this manuscript.
Mycoplasma contamination	Lines were routinely screened for mycoplasma by PCR after each thawing and prior to use in experiments.
Commonly misidentified lines<br>(See ICLAC register)	n/a

Animals and other research organisms

Policy information about studies involving animals; ARRIVE guidelines recommended for reporting animal research, and Sex and Gender in Research

Laboratory animals	mus musculus, strain: mixed C57bl6/ICR. Ages varied for experiments. For embryos, age was stated in Figure Legend. For postnatal analysis, mice were analyzed animals were between P30 and P60.

Wild animals	n/a
Reporting on sex	Data was collected using mice from both sexes. For postnatal analysis where sex was determined, we performed a post-hoc ANOVA analysis and determined that sex was not a confound in St18 (-/-) or St18 cKO mutant phenotypes.
Field-collected samples	n/a
Ethics oversight	All animal experiments were conducted in compliance with and pre-approval from Columbia University's Institutional Animal Care and Use Committee.

Note that full information on the approval of the study protocol must also be provided in the manuscript.
